# The lncRNA *lincNMR* regulates nucleotide metabolism via a YBX1 - RRM2 axis in cancer

**DOI:** 10.1038/s41467-020-17007-9

**Published:** 2020-06-25

**Authors:** Minakshi Gandhi, Matthias Groß, Jessica M. Holler, Si’Ana A. Coggins, Nitin Patil, Joerg H. Leupold, Mathias Munschauer, Monica Schenone, Christina R. Hartigan, Heike Allgayer, Baek Kim, Sven Diederichs

**Affiliations:** 1 Division of RNA Biology & Cancer, German Cancer Research Center (DKFZ), Heidelberg, Germany; 2 German Academic Exchange Service (DAAD), Bonn, Germany; Helmholtz International Graduate School for Cancer Research (HIGS), Heidelberg, Germany; Faculty of Biosciences, Heidelberg University, Heidelberg, Germany; 3 Department of Pediatrics, School of Medicine, Emory University, Atlanta, GA USA; 4 Department of Experimental Surgery—Cancer Metastasis, Medical Faculty Mannheim, Centre for Biomedicine and Medical Technology Mannheim, University of Heidelberg, Mannheim, Germany; 5 Broad Institute of MIT and Harvard, Cambridge, MA USA; 6 Center for Drug Discovery, Children’s Healthcare of Atlanta, Atlanta, GA USA; 7 Division of Cancer Research, Department of Thoracic Surgery, Medical Center, Faculty of Medicine, University of Freiburg, German Cancer Consortium (DKTK)—Partner Site Freiburg, Freiburg, Germany; 8 Present Address: Cold Spring Harbor Laboratory, Cold Spring Harbor, NY USA

**Keywords:** Cancer, DNA metabolism, Long non-coding RNAs

## Abstract

*Long intergenic non-coding RNA-Nucleotide Metabolism Regulator (lincNMR*) is a long non-coding RNA (lncRNA) which is induced in hepatocellular carcinoma. Its depletion invokes a proliferation defect, triggers senescence and inhibits colony formation in liver, but also breast and lung cancer cells. Triple-label SILAC proteomics profiles reveal a deregulation of key cell cycle regulators in *lincNMR*-depleted cells like the key dNTP synthesizing enzymes RRM2, TYMS and TK1, implicating *lincNMR* in regulating nucleotide metabolism. *LincNMR* silencing decreases dNTP levels, while exogenous dNTPs rescues the proliferation defect induced by *lincNMR* depletion. In vivo RNA Antisense Purification (RAP-MS) identifies YBX1 as a direct interaction partner of *lincNMR* which regulates RRM2, TYMS and TK1 expression and binds to their promoter regions. In a Chick Chorioallantoic Membrane (CAM) in vivo model, *lincNMR*-depleted tumors are significantly smaller. In summary, we discover a lincRNA, *lincNMR*, which regulates tumor cell proliferation through a YBX1-RRM2-TYMS-TK1 axis governing nucleotide metabolism.

## Abstract (web summary)

Despite some well-characterized functions in cancer, the impact of most long non-coding RNAs remains unknown. Here, the authors discover the lncRNA *lincNMR* which is upregulated in cancer and drives cell proliferation by interacting with YBX1 and controlling nucleotide metabolism.

## Introduction

A comprehensive landscape of the transcriptome has revealed that although over a 75% of human genome is transcribed, only about 2% of it encodes for proteins^1^. In recent years, over 10,000 long non-coding RNAs (lncRNAs) have been identified, and their number is ever increasing. Although lncRNAs were initially viewed as background noise from junk DNA, it is now evident that they play an important role in various biological processes, and their deregulation has been linked to various cancers including liver cancer^2,3^. LncRNAs act as drivers of carcinogenesis by regulating one or multiple hallmarks of cancer^4–6^. Several well-studied examples include lncRNAs regulating viability^7–9^, proliferation^10–12^, growth suppression^13,14^, migration^15–17^, angiogenesis^18,19^, and cellular immortality^20–22^. Detailed epigenetic, genomic, and transcriptional analyses have revealed that lncRNAs have cancer type-specific deregulation patterns^23^ and could be attractive molecules for therapeutic applications^24^. LncRNAs are heterogeneous molecules and play diverse functional roles by interacting with DNA, RNA, or proteins, such as recruitment of chromatin modifying complexes^25^ or transcription factors^26^, controlling mRNA stability^27^, and acting as competing endogenous RNAs^28^.

Liver cancer is the fourth leading cause of cancer-related mortalities worldwide in 2018, and is predicted to be sixth most commonly diagnosed cancer^29^. Of all liver cancers diagnosed, primary liver cancer called hepatocellular carcinoma (HCC) constitutes 75–85% of the cases with limited treatment options in advanced stages warranting further investigations. Notably, lncRNAs like *HULC*, *TERC*, and *HOTAIR* have also been implicated in hepatocarcinogenesis^28,30,31^.

In this study, we investigate lncRNAs induced in liver cancer patient samples derived from high-throughput RNA sequencing data and identify the lncRNA *lincNMR* (*long intergenic noncoding RNA–nucleotide metabolism regulator*) upregulated in HCC and impacting tumor growth in vivo. Its in-depth molecular characterization unravels its role in controlling nucleotide metabolism via interacting with YBX1 and regulating RRM2, TK1, and TYMS.

## Results

### *lincNMR* is upregulated in hepatocellular carcinoma

**Fig. 1:**
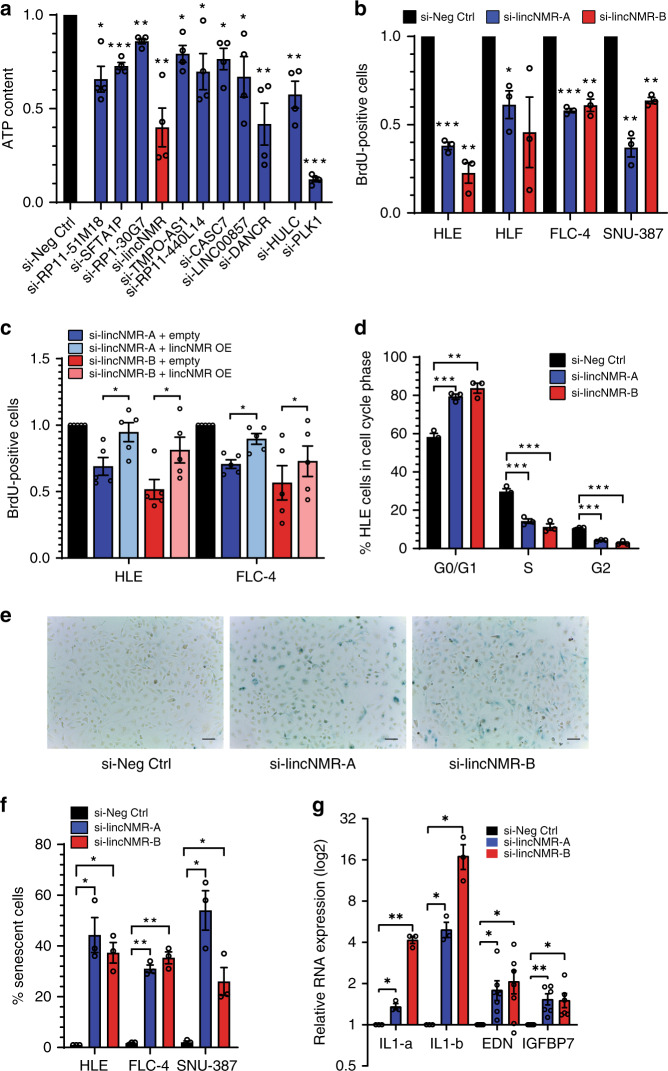
*LincNMR* depletion impairs cell viability, cell proliferation and induces senescence. **a** Impact of depletion of selected lncRNAs with 10 nM siPOOLs on cell viability as determined by CellTiter-Glo measuring the cellular ATP content after 72 h in HLE cells (*n* = 4). si-HULC and si-PLK1 served as positive controls. **b** Depletion of *lincNMR* with 10 nM of two independent siPOOLs invokes a strong proliferation defect in four liver cancer cell lines (HLE, HLF, FLC-4, and SNU-387) 72 h post transfection (*n* = 3). **c** Overexpression of *lincNMR* rescues the proliferation defect induced by *lincNMR* silencing in two different liver cancer cell lines, HLE and FLC-4. Data show BrdU assay readout at 72 h after *lincNMR* knockdown (KD), and 66 h after *lincNMR* overexpression (OE). Data shown are normalized to si-Neg Ctrl siPOOL transfected with empty vector pcDNA3.1 (*n* = 5). Significance was calculated by paired, two-tailed *t* test with *P < 0.05. **d** Silencing of *lincNMR* with 10 nM siPOOLs induces cell cycle arrest in the G0/G1 phase shown by flow cytometry 72 h post transfection in HLE cells (*n* = 3). Analysis was performed using FlowJo v10 software. **e** Representative microscopic images showing increased β-Gal activity indicating senescence in HLE cells 96 h post *lincNMR* knockdown with 10 nM siPOOLs (*n* = 3). The scale bar represents 100 µm. **f** Bar graph representing percent β-Gal-positive cells in multiple liver cancer cell lines after *lincNMR* depletion (*n* = 3). **g** Induction of senescence-associated secretary phenotype (SASP) markers IL-1a,IL-1b, EDN, and IGFBP7 determined by RT-qPCR at 72 h after *lincNMR* knockdown in HLE cells with 10 nM siPOOLs (*n* = 3). **a**–**d**, **f**, **g** Data represent mean, and error bars represent SEM. **a**, **b**, **d**, **f**, **g** Significance was calculated by unpaired, two-tailed *t* test with *P < 0.05; **P < 0.01; ***P < 0.001. **a**–**c**, **g** Data shown were normalized to negative control siPOOL.

To identify long noncoding RNAs (lncRNAs) deregulated in hepatocellular carcinoma (HCC), lncRNA expression was analyzed genome-wide based on the TCGA RNA sequencing dataset of liver cancer patients (tumor = 200 samples, normal = 50 samples). Out of 12,727 annotated lncRNAs in the TANRIC liver cancer dataset^32^, 217 lncRNAs were found to be significantly (*P* < 0.05) induced by at least fivefold (median tumor/normal). In total, 49 lncRNAs were selected based on their genomic location, repeat content, pseudogene content, and association with clinical properties, and their expression was validated in nine liver cancer cell lines (Supplementary Data 1). Nine expressed lncRNAs were further selected based on expression levels, coding potential, and novelty for RNAi-based phenotypic analysis using siPOOLs^33^. Among these candidates, the uncharacterized transcript RP6-65G23.3 yielded the strongest decrease of cell viability as determined by ATP content measurement in liver cancer cells, comparable with the effect of *PLK1*^34^ or *HULC*^35^ knockdown (Fig. 1a). For reasons described below, we named this lncRNA *lincNMR* (*long intergenic noncoding RNA–nucleotide metabolism regulator*).

### Basic characterization of *lincNMR*

*LincNMR* is a lncRNA transcribed from a bidirectional promoter in a head-to-head orientation on chromosome 14. Since the *lincNMR* transcript had never been studied, we defined its gene boundaries using rapid amplification of cDNA ends (RACE). 5′RACE identified a transcription start site (TSS) upstream of the current GENCODE annotation (Supplementary Fig. 1a). This finding was supported by RNA-Pol II Chip and switchgear TSS datasets (Supplementary Fig. 1b) corroborating the extended transcript identified in our 5′-RACE. 3′-RACE confirmed the previously annotated 3′-end of *lincNMR*, but also identified a second, less abundant isoform of *lincNMR*, including an additional internal exon (Supplementary Fig. 1c). Both isoforms have been deposited into Genbank with the accession numbers MK652436 and MK652437, respectively. Our 5′- and 3′-RACE results were supported by ENCODE/Cold Spring Harbor long RNA-Seq tracks from the ENCODE consortium^36^ (Supplementary Fig. 1a, c). Next, we analyzed the coding potential of *lincNMR* using scores from phyloCSF^37^ (Supplementary Fig. 1d) and the Coding Potential Calculator^38^ (Supplementary Fig. 1e). Both algorithms classified *lincNMR* as a noncoding transcript. We determined the copy number of *lincNMR* per cell with at least two to seven copies. Since the subcellular localization maybe linked to the biological function of a noncoding RNA^39,40^, we performed subcellular fractionation with fraction-specific controls *NEAT1* (chromatin fraction), *RNU-1* (nucleoplasmic fraction) and *DANCR* (cytoplasmic fraction). *LincNMR* predominantly localized with 60–70% to the cytoplasm, but also showed considerable abundance in the nucleoplasm (Supplementary Fig. 1f).

### *LincNMR* depletion affects cell proliferation and induces senescence

To elucidate the cellular function of *lincNMR*, we depleted *lincNMR* using two independent siPOOLs for additional specificity and to exclude any off-target effects observed with single siRNAs^33^ in multiple cancer cell lines. Both siPOOLs knocked down *lincNMR* efficiently in multiple liver (Supplementary Fig. 2a), breast (Supplementary Fig. 2b), and lung (Supplementary Fig. 2c) cancer cell lines. Since *lincNMR* knockdown decreased cell viability in liver cancer cells (Fig. 1a), cell proliferation was determined by performing BrdU incorporation assays. *LincNMR* silencing with two independent siPOOLs resulted in 30–80% decrease in cell proliferation in four liver cancer cell lines (HLE, HLF, SNU-387, and FLC-4) (Fig. 1b). Depletion of *lincNMR* also impaired cell proliferation in three breast (MCF-7, KPL-1, and T47D) (Supplementary Fig. 2d) and three lung (A549, NCI-H460, and NCI-H1299) cancer cell lines (Supplementary Fig. 2e). The overexpression of *lincNMR* rescued the proliferation defect caused by *lincNMR* depletion attesting to its specificity (Fig. 1c). Furthermore, a cell cycle analysis using flow cytometry confirmed an increase of cells in the G0/G1 phase of the cell cycle after depletion of *lincNMR* in multiple cell lines (Figs. 1d and Supplementary S2f, g).

The arrest of cells in the G0 / G1 phase prompted us to evaluate the induction of senescence. Depletion of *lincNMR* triggered senescence in three liver cancer cells with two independent siPOOLs as evident by β-GAL-positive blue cells in SA-β-GAL assay (Fig. 1e, f). The induction of senescence was supported by the induction of the pro-inflammatory cytokines IL-1a and IL-1b, which are bona fide markers of the senescence-associated secretary phenotype (SASP) as well as the senescence-associated proteins EDN and IGFBP7 (Fig. 1g). The induction of senescence was largely independent of the expression of p53 or pRB (Supplementary Fig. 2h) and also did not have gross consistent effects on p53 or pRB signaling (Supplementary Fig. 2i). *LincNMR* knockdown neither had a consistent impact on apoptosis (Supplementary Fig. 3a) nor did its overexpression affect proliferation in cancer cells (Supplementary Fig. 3b).

### *lincNMR* is induced in multiple cancer entities

**Fig. 2:**
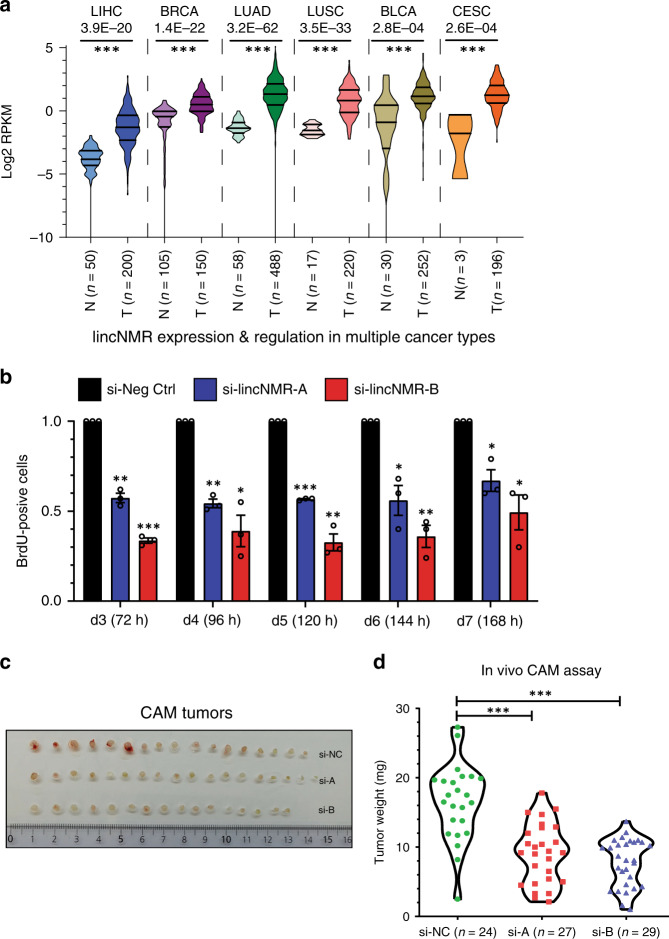
*lincNMR* is induced in multiple cancer types and affects tumor growth in vivo. **a** *LincNMR* is expressed and regulated in multiple cancer types (*N* = normal, *T* = tumor; data obtained from TCGA data portal). LIHC = hepatocellular carcinoma (*N* = 50, *T* = 200), BRCA = breast cancer (*N* = 105, *T* = 105), LUAD = lung adenocarcinoma (*N* = 58, *T* = 488), LUSC = lung squamous cell carcinoma (*N* = 17, *T* = 220), BLCA = bladder cancer (*N* = 30, *T* = 252), CESC = cervical serous carcinoma (*N* = 3, *T* = 196). Data are represented as log2 RPKM. **b** Timecourse of BrdU incorporation assays in HLE cells showing induction and maintenance of proliferation inhibition up to 168 h post *lincNMR* knockdown with 10 nM siPOOLs (*n* = 3). Data are normalized to negative control siPOOL. Data represent mean, and error bars represent SEM. **c** Chick chorioallantoic membrane (CAM) assay: picture showing CAM-harvested tumors formed from HLE cells transfected with *lincNMR* or control siPOOLs. Tumors were harvested on day 6. *LincNMR*-depleted cells gave rise to smaller tumors (the number of chick embryos used in total: si-negative control = 24, si-*lincNMR*-A = 27, si-*lincNMR*-B = 29). **d** Quantification of tumors harvested from CAM assay: decreased tumor weight in *lincNMR*-depleted tumors compared to the control group. **a**, **b**, **d** Significance was calculated by unpaired, two-tailed *t* test with *P < 0.05; **P < 0.01; ***P < 0.001.

In line with the impact of *lincNMR* knockdown in cell lines from different tumor entities, *lincNMR* was also significantly induced between tumor and normal tissues across multiple cancer types, including breast invasive carcinoma, lung adenocarcinoma, lung squamous cell carcinoma, bladder urothelial carcinoma, and cervical squamous cell carcinoma (Fig. 2a). In addition, *lincNMR* was also expressed in a broad panel of 73 cell lines derived from different tumor entities or normal tissues (Supplementary Fig. 3c).

### *lincNMR* depletion leads to decreased tumor growth in vivo

To test the impact of *lincNMR* knockdown on tumor growth in vivo, we turned to the chicken chorioallantoic membrane (CAM) model for in vivo xenograft experiments following the ethical responsibility aiming to replace, reduce or refine (3R) the use of animal models for research purposes. Knockdown efficiency (Supplementary Fig. 3d) and the presence of the growth-inhibitory phenotype (Fig. 2b) were confirmed for the duration of the CAM assay until 168 h (day 7). After seeding *lincNMR*-silenced or control-treated cells onto the CAM, tumors were harvested, measured, and weighed. Tumors derived from *lincNMR*-depleted cells were significantly smaller in size than tumors derived from the control group (Fig. 2c). The tumor weight was also significantly reduced in *lincNMR*-depleted tumors in comparison with the control group (Fig. 2d).

### *lincNMR* binds to YBX1 protein, which regulates cell survival

**Fig. 3:**
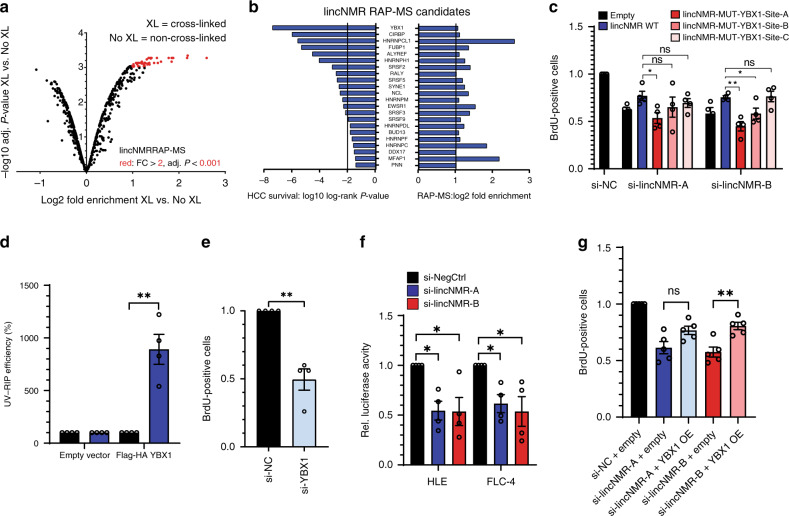
*lincNMR* directly binds to and controls YBX1. **a** Volcano plot depicting total candidates (*n* = 701) identified by *lincNMR* in vivo RNA antisense purification followed by mass spectrometry (RAP-MS). In total, 48 candidates highlighted in red were selected for fold enrichment (FC > 2) and adjusted *P*-value of enrichment (*P* < 0.001). XL: cross link. Significance was calculated by moderated *t* test. **b** Interaction candidates identified by in vivo RAP-MS selected for fold enrichment in cross linked over non-cross linked samples (FC > 2), adjusted *P *- value of enrichment (*P* < 0.001), and sorted for log-rank *P *- value in liver cancer patient survival (based on TCGA data). **c** While the overexpression of wild-type (WT) *lincNMR* partially rescues the decreased cell proliferation caused by *lincNMR* knockdown, a *lincNMR* mutant with a mutation in the first (A) of three (A, B, C) putative YBX1-binding sites is not capable of rescuing cell proliferation in HLE cells at 72 h post *lincNMR* depletion (assayed by BrdU incorporation, *n* = 4). **d** Determination of RNA pull down efficiency in UV–RIP by RT-qPCR validating the interaction between YBX1 and *lincNMR* in HLE cells (*n* = 4). Data shown are normalized to SRSF4 as a negative control gene and control vector. **e** Silencing YBX1 with 10 nM siPOOL imparts a proliferative disadvantage to HLE cells determined 72 h post transfection (*n* = 4). Data shown are normalized to negative control siPOOL. **f** Dual-luciferase assay for the transactivational activity of YBX1, which unravels the inhibition of YBX1 by *lincNMR* silencing. Data shown are the normalized ratios of YBX1-dependent Firefly luciferase activity divided by Renilla luciferase used for standardization after depletion of *lincNMR* with 10 nM siPOOLs in HLE and FLC-4 cells (*n* = 4). **g** Overexpression of YBX1 partially rescues the proliferation defect induced by *lincNMR* depletion in HLE cells assayed by BrdU cell proliferation assay (72 h depletion of *lincNMR* and 66 h overexpression of YBX1). Data shown are normalized to empty vector transfected with negative control siPOOL (*n* = 5). **c**–**g** Data represents mean and error bars represent SEM. Significance was calculated by unpaired, two-tailed *t* test with *P < 0.05; **P < 0.01; ***P < 0.001.

In order to gain insight into the molecular function and protein interaction partners of *lincNMR*, we performed in vivo RNA antisense purification (RAP-MS)^41,42^. After cross linking RNA and protein in vivo, we used biotinylated DNA oligos complementary to the *lincNMR* sequence to pull down *lincNMR* and its associated protein-binding partners from HLE cell lysates (Supplementary Fig. 4a). Pull down efficiency was confirmed by RT-qPCR with three different *lincNMR* amplicons, whereas *PPIA* and *GAPDH* mRNAs were used as negative controls (Supplementary Fig. 4b). Mass spectrometry analysis of proteins cross linked to and pulled down with *lincNMR* compared with non-cross linked lysates identified 701 proteins in total (Fig. 3a), with 48 proteins or isoforms enriched at least twofold and with an adjusted *P*-value < 0.001 (Supplementary Data 2). To further select relevant interaction partners linked to HCC, we analyzed their correlation to survival in HCC (Fig. 3b). In total, 22 candidates were significantly associated with the overall survival of liver cancer patients (Supplementary Data 2). YBX1 emerged as a potential binding partner with strongest and significant correlation to survival in liver cancer patients (Fig. 3b; Supplementary Fig. 4c). In addition, we searched for predicted RBP binding sites in the target genes using RBPmap^43^ and found three YBX1 sites and two SRSF3 sites (*P* < 0.001) in the *lincNMR* transcript. Mutation of the first of the three predicted consensus YBX1 sites in *lincNMR* (Supplementary Fig. 4d) abrogated the proliferation rescue of wild-type *lincNMR* (Fig. 3c). UV–RIP followed by RT-qPCR validated the interaction between *lincNMR* RNA and YBX1 protein. After immunoprecipitation of Flag-HA-tagged YBX1 (Supplementary Fig. 4e), *lincNMR* was enriched in the YBX1 pulldown compared with the negative control SRSF4 (Fig. 3d). In addition, biotinylated *lincNMR* RNA pulled down endogenous YBX1 protein in an in vitro RNA-affinity purification (Supplementary Fig. 4f).

In addition to the significant association of high YBX1 expression with survival in hepatocellular carcinoma patients (Supplementary Fig. 4c), knockdown of YBX1 impaired cell proliferation by 50% (Fig. 3e) resembling the effect of *lincNMR* knockdown. *LincNMR* levels were decreased by 49% after YBX1 depletion (Supplementary Fig. 4g). Vice versa, YBX1 protein levels were decreased by ~20% when *lincNMR* was silenced by two independent siPOOLs (Supplementary Fig. 4h). We also found a significant positive correlation between *lincNMR* and *YBX1* mRNA in HCC patient samples (Supplementary Fig. 4i).

The direct interaction of *lincNMR* and YBX1 protein prompted us to assess the activity of YBX1 upon *lincNMR* depletion using luciferase assays for YBX1 transactivational activity. Knockdown of *lincNMR* significantly decreased the YBX1 activity in two independent liver cancer cell lines (Fig. 3f). In turn, overexpression of YBX1 partially rescued the proliferation deficit caused by *lincNMR* knockdown (Fig. 3g). Together, these data show that *lincNMR* interacts with and regulates YBX1, YBX1 mimics the impact of *lincNMR* on cell proliferation, and the regulation of *lincNMR* by YBX1 generates a feedforward loop leading to the correlation of expression in liver cancer.

### *lincNMR* and YBX1 share target genes in nucleotide metabolism

To investigate the impact of *lincNMR* on the cellular proteome, we employed a triple-label stable isotope labeling by amino acids in cell culture (SILAC) approach (schematic Supplementary Fig. 5a). SILAC ratios were used for analysis comparing siPOOLs targeting *lincNMR* to negative control siPOOL (si-lincNMR-A/si-Neg Ctrl = M/L and si-lincNMR-B/si-Neg Ctrl = H/L). A correlation analysis served as a quality control on the complete dataset: a significant correlation was observed across three biological replicates and in between both siPOOLs targeting *lincNMR* with an average correlation coefficient of *R* = 0.74 (Supplementary Fig. 5b). Notably, the correlation between replicates of the same siPOOL was slightly higher (range 0.69 – 0.87) than between two different siPOOLs (range 0.43–0.54), which may point towards sequence-mediated off-target effects, but still indicates the high specificity and correlation (*P* < 10^−100^) of the complex siPOOLs of 30 siRNAs compared with an average correlation of only 0.07 between individual siRNAs or lower complexity pools^44^.

**Fig. 4:**
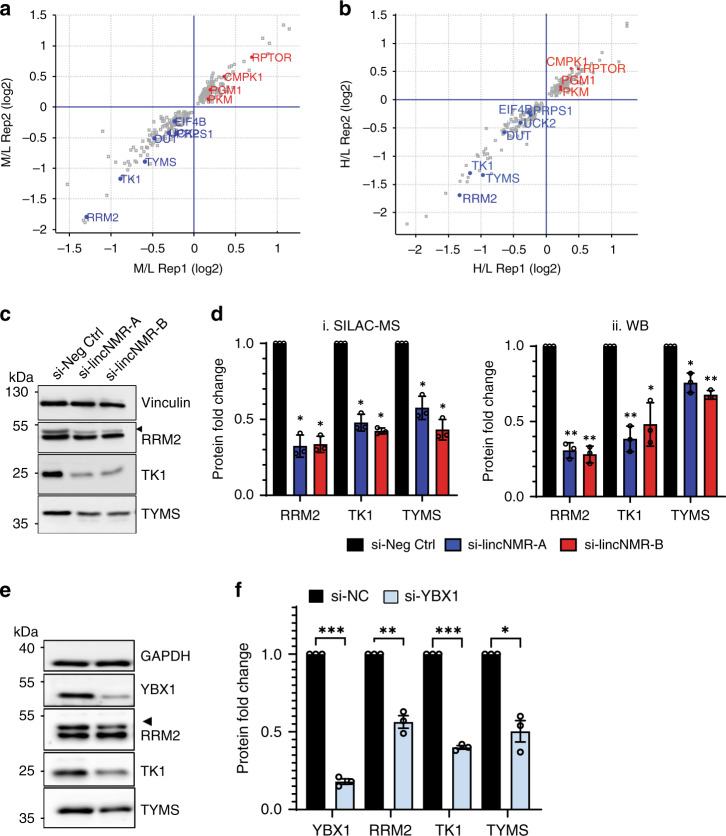
*lincNMR* depletion downregulates key dNTP metabolism enzymes. **a** Triple-label SILAC-MS: scatter plot showing normalized M/L ratios representing deregulated proteins 48 h after *lincNMR* knockdown in replicates 1 and 2 with 10 nM siPOOLs in HLE cells (M/L = si-lincNMR-A/si-Neg Ctrl). **b** Triple-label SILAC-MS: scatter plot showing normalized H/L ratios representing deregulated proteins 48 h after *lincNMR* knockdown in replicates 1 and 2 with 10 nM siPOOLs in HLE cells (H/L = si-lincNMR-B/si-Neg Ctrl). **c** Western blot validation of SILAC-MS data depicting downregulation of RRM2, TK1, and TYMS proteins in HLE cells 72 h after *lincNMR* knockdown with 10 nM siPOOLs (*n* = 3). Vinculin was used as a loading control. **d** Quantitative comparison of SILAC-MS (i) and western blot (ii) results confirming consistent downregulation of RRM2, TK1 and TYMS (*n* = 3). log2 fold change was calculated and normalized to negative control siPOOL. **e** YBX1 silencing inhibits the expression of RRM2, TK1, and TYMS at 72 h post transfection with 10 nM siPOOL in HLE cells documented by western blotting (*n* = 3). GAPDH was used as a loading control. **f** Quantification of western blot from (**e**): protein fold change was calculated by normalizing the RRM2, TK1, and TYMS signal to loading control GAPDH and to negative control siPOOL (*n* = 3). **a**, **b** Data represent log2 fold change normalized to negative control siPOOL from two independent replicates. Highlighted proteins represent key deregulated players in purine and pyrimidine metabolism according to KEGG pathway annotations. Color key: red = upregulated proteins; blue = downregulated proteins. **d**, **f** Data represent mean, and error bars represent SEM. Significance was calculated by unpaired, two-tailed t test with *P < 0.05; **P < 0.01; ***P < 0.001.

**Fig. 5:**
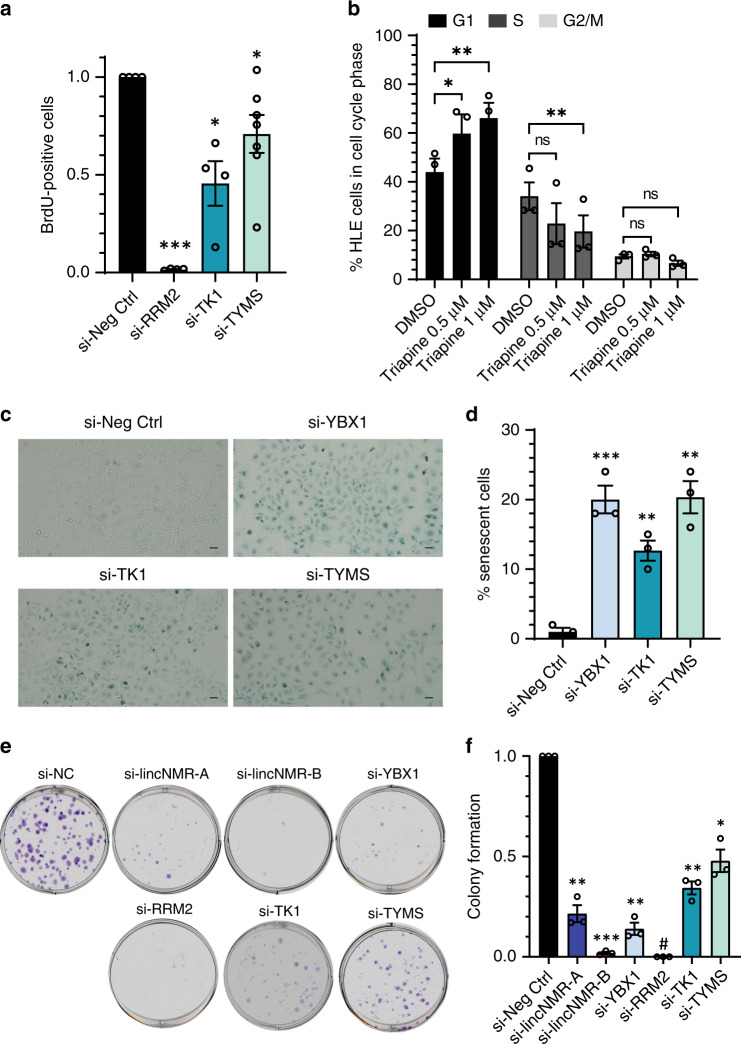
Silencing *YBX1, RRM2, TK1*, or *TYMS* mimics the phenotype of *lincNMR* depletion. **a** Depletion of RRM2, TK1, and TYMS invokes a strong proliferation defect 72 h after knockdown using 10 nM siPOOLs targeting RRM2, TK1, or TYMS in HLE cells in at least three biological replicates. **b** The RRM2 inhibitor Triapine (0.5 µM and 1 µM) induces cell cycle arrest in the G0 / G1 phase at 72 h post treatment in HLE cells. DMSO was used as a control. Data analysis was performed using the cell cycle analysis platform in the FlowJo software v10 (*n* = 3). **c** Representative microscopic images showing increased β-Gal activity indicating induction of senescence in HLE cells at 96 h post knockdown with 10 nM of the respective siPOOLs (*n* = 3). The scale bar represents 100 µm. **d** SA-β-Gal assay quantification: bar graph representing percent β-Gal-positive cells after depletion of YBX1, TK1, and TYMS in HLE cells (*n* = 3). RRM2-depleted cells were abolished, and hence not available for analysis. **e** Representative images showing the impact of silencing of *lincNMR*, YBX1, RRM2, TK1, and TYMS on colony-forming efficiencies at 10 days after transfection using 10 nM siPOOLs in HLE cells (*n* = 3). **f** Quantification of colony formation after knockdown represented relative to the negative control siPOOL (*n* = 3). #: Following RRM2 knockdown, no colonies were growing (0) and hence, no statistical significance could be calculated. **a**, **b**, **d**, **f** Data represent mean, and error bars represent SEM. Significance was calculated by unpaired, two-tailed *t* test with *P < 0.05; **P < 0.01; ***P < 0.001.

For 242 candidates (*P*-value <0.001) deregulated by both *lincNMR*-targeting siPOOLs in the same direction, a Panther Overrepresentation Analysis with FDR correction was performed using Reactome and PANTHER pathway datasets. This revealed a significant enrichment of key terms like “G1 / S transition” and “Cell Cycle Checkpoints” as well as “De novo pyrimidine deoxyribonucleotide biosynthesis” matching the phenotype observed after *lincNMR* depletion (Supplementary Fig. 5c). Among the strongly downregulated proteins upon *lincNMR* knockdown, we identified RRM2 (si-lincNMR-A = −68%, si-lincNMR-B = −67%), TK1 (si-lincNMR-A = −52%, si-lincNMR-B = −58%), and TYMS (si-lincNMR-A = −43%, si-lincNMR-B = −57%) and other key enzymes in nucleotide metabolism pathways (Fig. 4a, b), which were also part of the enriched gene ontology terms (Supplementary Fig. 5c). This decrease of RRM2, TK1, and TYMS proteins after *lincNMR* depletion was validated by western blotting (Fig. 4c) in good accordance with the independent triple-label SILAC-MS approach (Fig. 4d). In addition, a decrease of *RRM2*, *TK1*, and *TYMS* mRNAs was also observed after *lincNMR* depletion with both siPOOLs (Supplementary Fig. 5d). Since we identified *lincNMR* as a regulator of the transcription factor YBX1, we tested whether also YBX1 depletion would affect RRM2, TK1, and TYMS expression levels. Indeed, loss of YBX1 induced a significant decrease of RRM2, TK1, and TYMS protein levels (Fig. 4e, f) establishing the *linc*NMR–YBX1–RRM2 / TK1 / TYMS axis. Depletion of RRM2, TK1, and TYMS with siPOOLs significantly impaired cell proliferation mimicking the phenotype observed after *lincNMR* depletion (Fig. 5a) with a knockdown efficiency in the range of 95–99% (Supplementary Fig. 5e). Similarly, the RRM2 inhibitor triapine^45^ induced an arrest of cell cycle progression in the G1 phase similar to *lincNMR* knockdown (Fig. 5b).

Knockdown of YBX1, TK1, or TYMS also significantly induced cellular senescence (Fig. 5c, d), further phenocopying the *lincNMR* knockdown. Knockdown of *lincNMR*, YBX1, TK1, and TYMS also significantly inhibited the colony-formation capacity of liver cancer cells (Fig. 5e, f). After the knockdown of RRM2, no cells were left precluding quantification of senescence or colony formation.

Next, we found *RRM2*, *TK1*, and *TYMS* to be strongly and significantly induced by about ten-, six- and fourfold, respectively, in liver cancer patient datasets from TCGA (Supplementary Fig. 6a–c). Furthermore, all three target genes, *RRM2*, *TK1*, and *TYMS* were significantly associated with poor survival in HCC patients (Supplementary Fig. 7a–c). The *lincNMR* expression level significantly positively correlated with *RRM2*, *TK1*, and *TYMS* mRNA levels in HCC patient samples (Supplementary Fig. 7d–f), further corroborating the strong link between *lincNMR*, these three regulators of nucleotide metabolism, and liver cancer.

**Fig. 6:**
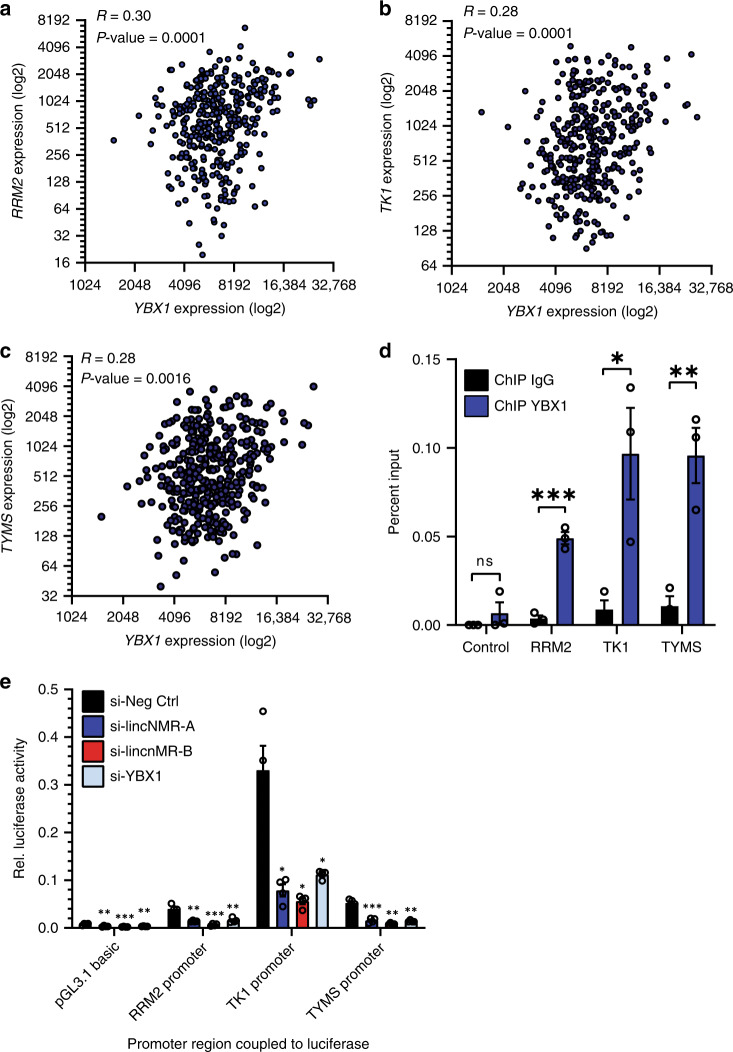
*YBX1* correlates with, binds to and activates the promoters of *RRM2*, *TK1*, and *TYMS*. **a** YBX1 mRNA expression significantly and positively correlates with RRM2 mRNA expression in *n* = 374 hepatocellular carcinoma patient samples (TCGA LIHC, indicated: log2 expression). Significance was calculated by unpaired, two-tailed *t* test. **b** YBX1 mRNA expression significantly and positively correlates with TK1 mRNA expression in *n* = 374 hepatocellular carcinoma patient samples (TCGA LIHC, indicated: log2 expression). Significance was calculated by unpaired, two-tailed *t* test. **c** YBX1 mRNA expression significantly and positively correlates with TYMS mRNA expression in *n* = 374 hepatocellular carcinoma patient samples (TCGA LIHC, indicated: log2 expression). Significance was calculated by unpaired, two-tailed *t* test. **d** The binding of YBX1 to the promoters of *RRM2*, *TK1*, and *TYMS* was determined by chromatin immunoprecipitation (ChIP) in HLE cells. Bar graph represents qPCR data after YBX1 ChIP compared with IgG as a negative control (*n* = 3). Data represent mean, and error bars represent SEM. Significance was calculated by unpaired, two-tailed *t* test where *P < 0.05; **P < 0.01; ***P < 0.001. **e** Luciferase assays show decreased transactivation of *RRM2*, *TK1*, and *TYMS* promoter regions after *lincNMR* or YBX1 silencing with 10 nM siPOOLs in HLE cells (*n* = 4). Data shown are ratios of firefly luciferase divided by Renilla luciferase used for standardization. Data represent mean, and error bars represent SEM. Significance was calculated by unpaired, two-tailed *t* test where *P < 0.05; **P < 0.01; ***P < 0.001.

Also, the expression of *YBX1* mRNA significantly positively correlated with *RRM2*, *TK1*, and *TYMS* mRNA expression in 374 human hepatocellular carcinoma samples (TCGA, Fig. 6a–c). The YBX1 protein interacted with the promoter regions of the *RRM2*, *TK1*, and *TYMS* genes as revealed by chromatin immunoprecipitation (ChIP, Fig. 6d). Lastly, we performed luciferase assays with the promoter regions of *RRM2*, *TK1*, and *TYMS* fused to firefly luciferase. Knockdown of *lincNMR* or YBX1 significantly decreased the luciferase activity for all three promoters (Fig. 6e), further corroborating the regulatory interaction of *lincNMR* and YBX1 with *RRM2*, *TK1*, and *TYMS*.

### Depletion of *lincNMR* leads to reduced dNTP levels

**Fig. 7:**
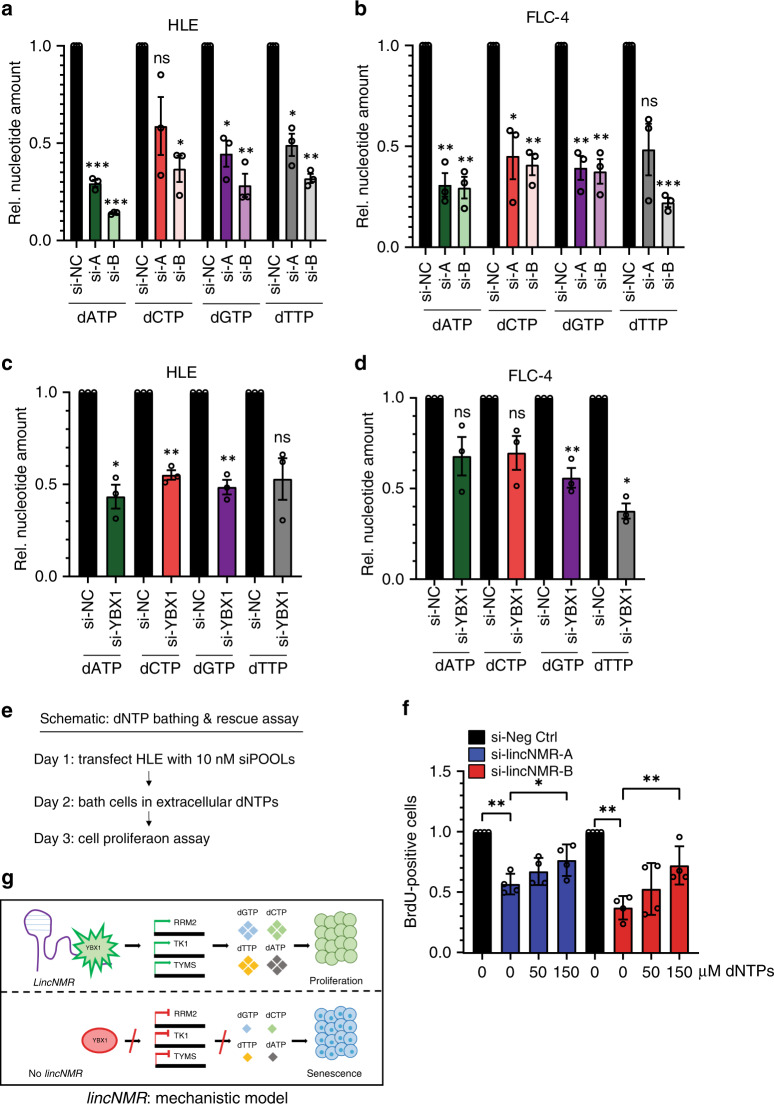
*LincNMR* depletion reduces dNTP levels while supplying dNTPs rescues the *lincNMR* phenotype. **a** Quantification of dNTP levels uncovers that the depletion of *lincNMR* with 10 nM siPOOLs leads to strong downregulation of dATP, dCTP, dGTP, and dTTP in HLE cells (*n* = 3). **b** Quantification of dNTP levels uncovers that the depletion of *lincNMR* with 10 nM siPOOLs leads to strong downregulation of dATP, dCTP, dGTP, and dTTP in FLC-4 cells (*n* = 3). **c** Quantification of dNTP levels uncovers that the depletion of YBX1 with 10 nM siPOOLs leads to downregulation of dATP, dCTP, dGTP, and dTTP in FLC-4 cells (*n* = 3). **d** Quantification of dNTP levels uncovers that the depletion of YBX1 with 10 nM siPOOLs leads to downregulation of dATP, dCTP, dGTP, and dTTP in FLC-4 cells (*n* = 3). **e** Schematic outline of dNTP bathing & rescue assay. **f** Supplying dNTPs rescues dose-dependently the proliferation decrease caused by *lincNMR* silencing determined by a BrdU incorporation assay. HLE cells were reverse transfected with 10 nM of the respective siPOOLs and bathed in increasing concentrations (0–150 µM) of extracellular pools of dNTPs 24 h post lincNMR depletion (*n* = 4). Data shown represent the results of the cell proliferation assay normalized to negative control siPOOL with the respective dNTP concentration. **g** Proposed model of *lincNMR* molecular mechanism. **a**–**d**, **f** Data shown are normalized to negative control siPOOL. Data represent mean, and error bars represent SEM. Significance was calculated by unpaired, two-tailed *t* test with *P < 0.05; **P < 0.01; ***P < 0.001.

Since dNTP-synthesizing enzymes were downregulated by the knockdown of *lincNMR*, we further investigated whether the levels of dNTPs were accordingly affected. All four dNTPs, dATP, dCTP, dGTP, and dTTP, were significantly downregulated after *lincNMR* depletion with two independent siPOOLs in two independent cell lines (Fig. 7a, b). The knockdown of YBX1 recapitulated this phenotype also leading to decreased dNTP levels in two cell lines (Fig. 7c, d).

### dNTPs rescue the *lincNMR* proliferation phenotype

Importantly, supplying exogenous dNTP pools by bathing the cells rescued the impact of *lincNMR* knockdown on cell proliferation by preventing this phenotype (Fig. 7e, f). This effect was dose-dependent and statistically significant, illustrating the essential role of nucleotide metabolism in the pro-proliferative function of *lincNMR*.

In summary, their regulation, their association with survival, and their correlation of expression links *lincNMR*, YBX1, RRM2, TK1, and TYMS to liver cancer and to each other, respectively. *LincNMR* affects cell viability, proliferation, senescence, colony formation, and tumor growth in vivo. The interactor YBX1 and its targets RRM2, TK1, and TYMS mimic the phenotypes of *lincNMR*. At the molecular level, these data suggest a model (Fig. 7g) in which the lncRNA *lincNMR* binds to YBX1, increases its activity resulting in the upregulation of the enzymes RRM2, TK1, and TYMS, which mediate an increase in nucleotide metabolism. In turn, *lincNMR* depletion causes a decrease of dNTPs leading to cellular senescence.

## Discussion

In this study, we identified a lncRNA – *lincNMR*, a first lncRNA to regulate nucleotide metabolism in cancer cells. Silencing of *lincNMR* leads to impaired cell proliferation and colony formation, induction of a G0 / G1 phase cell cycle arrest, deregulation of nucleotide metabolism, and eventual induction of senescence in multiple cancer cell lines. *LincNMR* overexpression rescues the proliferation defect pointing toward the specificity of the knockdown.

Cellular senescence is defined by an irreversible cell growth arrest. While cells undergoing senescence do not replicate, they remain metabolically active and undergo alterations in cell metabolism pathways, including nucleotide, glucose, mitochondrial, and lipid metabolism^46^.

Nucleotide pools are essential for a multitude of biological processes, and their synthesis is carefully regulated during cell proliferation. dNTP pools are synthesized de novo or via the salvage pathway^47^. The rate-limiting step in the synthesis of dNTP pools is the reduction of ribonucleoside di- or tri-phosphates (NDPs / NTPs) to deoxyribonucleotide di- or tri-phosphates (dNDPs / dNTPs) by ribonucleotide reductase (RNR)^48,49^. Deregulation of nucleotide metabolism has been reported to play a pathogenic role in various diseases, including cancer^50,51^.

In our triple-label SILAC-MS, knockdown of *lincNMR* leads to strong downregulation of key enzymes essential for dNTP biosynthesis — RRM2 (ribonucleotide reductase subunit 2), TK1 (thymidine kinase), and TYMS (thymidylate synthetase) among other cell cycle regulators and proliferation markers. These are also induced in HCC, associated with poor survival, and correlated with *lincNMR* expression, and their knockdown phenocopies the effect of *lincNMR* on cell proliferation, senescence, and colony formation.

Consequently, all four dNTPs are depleted upon *lincNMR* knockdown, consolidating its role in the nucleotide metabolism axis. Our findings are in line with previous studies, finding levels of all four dNTPs significantly decreased during OIS^50–52^ due to a suppression of RRM2 as a driver and not as an effect of cell cycle exit^51^. Depleting p53 or pRB, two key factors in senescence, does not affect *lincNMR*-controlled senescence in good accordance with a previous study showing that senescence induced by RRM2 depletion is independent of p53 and pRB^51^.

Importantly, bathing cells in exogenous dNTP pools dose-dependently rescues the proliferation phenotype caused by *lincNMR* knockdown in two liver cancer cell lines, which is in good accordance with previous studies stating that increasing dNTP levels by RRM2 overexpression or exogenous nucleoside supply overcomes aberrant DNA replication, DNA damage, and senescence induced by oncogenic RAS or BRAF^51,52^. Ectopic co-expression of TYMS and RRM2 also suppresses OIS in normal human fibroblasts^52,53^. TYMS and RRM2 are suppressed in c-MYC-depleted melanoma cells undergoing OIS, and this senescent effect is rescued by overexpression of TYMS and RRM2 or by addition of deoxyribonucleosides^54^.

To gain an insight into the molecular mechanism, we identified YBX1 as a direct binding partner of *lincNMR* in vivo and in vitro. YBX1 is a transcription factor and also binds to lncRNAs linked to cancer^55–58^. YBX1 drives tumorigenicity and invasiveness of melanoma cells and its expression represents a negative prognostic factor in primary melanoma patients^59^. YBX1 expression correlates with poor outcomes in breast cancer patients^60^. Accordingly, we find high YBX1 expression correlated with poor overall survival in liver cancer, as well. *LincNMR* and *YBX1* mRNA expression significantly correlate in liver cancer patient samples as well as with *RRM2*, *TK1*, and *TYMS* mRNA expression. Importantly, YBX1 depletion mimics the *lincNMR* depletion phenotype in decreasing cell proliferation, inducing senescence, diminishing colony formation, affecting RRM2, TK1, and TYMS levels, decreasing dNTP levels and YBX1 also partially rescues the growth-inhibitory effect of *lincNMR* knockdown. YBX1 binds to the promoter regions of *RRM2*, *TK1*, and *TYMS. LincNMR* controls the transactivational activity of YBX1— hence, we report a role for the *linc*NMR–YBX1 axis in regulating nucleotide metabolism in liver cancer cells. Nonetheless, future studies will unravel whether *lincNMR* primarily acts via recruiting, regulating, or activating YBX1, and these data also do not exclude that *lincNMR* may have additional functions and relevant interactors.

While the findings that *lincNMR* RNA interacts with YBX1 protein, that it shares the same target genes RRM2, TK1, and TYMS, that *lincNMR*, YBX1, RRM2, TK1, and TYMS share the same loss-of-function phenotype regarding cell proliferation, senescence, and colony formation, that YBX1 expression or exogenous dNTPs rescue the proliferation defect caused by *lincNMR* depletion, and that *lincNMR* and YBX1 depletion reduces the transactivation of RRM2, TK1, and TYMS promoter fragments in luciferase assays, arguing in favor of a direct effect of the *lincNMR*–YBX1 axis, future research may also investigate secondary effects by cell cycle disturbance or by an impact on YBX1 regulation.

The copy number with a conservatively approximated minimum range of two to seven copies per cell on average classifies *lincNMR* as a moderately expressed lncRNA while other functional lncRNAs show much lower copy numbers^61^. On the one hand side, this may already be sufficient to recruit YBX1 to specific loci in the genome — on the other hand, this is likely an underestimation since the comparison is necessarily done to plasmid DNA, so the efficiency of RNA isolation and reverse transcription is assumed to be quantitatively complete. Furthermore, the copy number of *lincNMR* could vary between different cell states (e.g., cell cycle, heterogeneity) and hence reach higher numbers for activity.

Depletion of *lincNMR* not only reduces proliferation in liver cancer but also impairs proliferation in multiple breast and lung cancer cell lines. In addition, it is overexpressed in multiple cancer entities like liver, lung, breast, bladder, and cervical cancer making it a likely broadly relevant oncogenic lncRNA. Xenograft experiments using *lincNMR*-depleted cells in the CAM model reveal the impact of *lincNMR* on tumor size in vivo potentially implicating it as a therapeutic target in the future.

Notably, targeting nucleotide metabolism via ribonucleotide reductase inhibitors has been identified as a promising therapeutic strategy in multiple cancer types. Ribonucleotide reductase (RNR) inhibitors show promise in the clinic for treating multiple cancer types with some even serving as a first-line cancer treatment. Gemcitabine is the first nucleoside analog clinically approved, and it continues to be a frontline therapy against pancreatic, bladder, and lung cancer^62^. Clofarabine is the second approved drug targeting refractory pediatric leukemia^63,64^. A combination therapy with gemcitabine, clofarabine, and carboplatin significantly improves progression-free survival of patients with platinum-sensitive recurrent ovarian cancer^65^. Another RNR inhibitor, hydroxyurea (HU), is used in treatment of AML, CML, and glioblastomas^66–68^.

Overall, our study identified a tumor-promoting lincRNA — *lincNMR *— and unveils its mechanism along a YBX1–RRM2 / TK1 / TYMS axis in regulating nucleotide metabolism and governing the cancer cell fate between proliferation and senescence.

## Methods

### Cell culture

Liver cancer cell lines (HLE, HLF, FLC-4, and SNU-387) used in this study were kindly provided by Dr. Kai Breuhahn (Institute of Pathology, University Heidelberg, Heidelberg Germany). Lung cancer cell lines (A549, NCI-H1299, and NCI-H460) and breast cancer cell lines (MCF-7, KPL-1, and T47D) were purchased from ATCC. Liver cancer cells were cultured in RPMI-1640 (Sigma-Aldrich, R8758) with 10% FBS. Lung and breast cancer cell lines were cultured in DMEM (Sigma-Aldrich, D5671) supplemented with 10% FBS. All cell lines were cultured in a cell culture incubator at 37 °C and 5% CO_2_ without addition of any antibiotics. Cell lines were periodically tested at a 3-month interval for mycoplasma contamination using a PCR-based detection kit (PromoCell, PK-CA91-1048). All cell lines used in this study were verified using cell authentication services from Multiplexion, Heidelberg, Germany^69^.

### siPOOL and plasmid transfections

siPOOLs were obtained as complex pools of 30 siRNAs targeting the same gene from siTOOLs Biotech GmbH, Martinsried, Germany, to minimize off-target effects^33^. siPOOLs were reverse transfected using Lipofectamine RNAiMAX transfection reagent (Life Technologies, 133778150) with RPMI-1640 medium. Plasmids were transfected using a forward transfection protocol by using TurboFect transfection reagent (Fischer Scientific, R0531) and Opti-MEM (Gibco, 31985054). Transfections were performed according to the manufacturer’s recommendations. Sequences of all siPOOLs used in this study are provided in Supplementary Data 3.

The double knockdown of TP53 and pRb was performed as described^70^. siPOOL sequences are listed in Supplementary Data 3.

### RNA isolation, reverse transcription, and quantitative PCR

Cells were lysed in Trizol (Sigma, T9424-200ML), RNA isolation and DNase I (Roche, 4716728001) digestion were performed as per the manufacturer’s instructions. RNA isolation for in vivo RAP-MS and UV - RIP qPCR experiments was performed as per the manufacturer’s protocol using the miRNeasy Mini kit (Qiagen, 217004), and DNase digestion was performed using the TURBO DNA*free* kit (Life Technologies, AM1907).

In total, 1 µg of RNA was reverse transcribed using random hexamer primers with RevertAid reverse transcriptase (ThermoFisher, EP0442). cDNA was diluted 1:40 with DNase- and RNase-free water, and 4 µl was used in a qPCR reaction. RT-qPCR was performed using Power SYBR Green PCR Master Mix (ThermoFisher, 4367659) in an Applied Biosystems StepOne Plus thermal cycler with holding stage of 95 °C for 10 min followed by 40 cycles of 95 °C for 15 s and 60 °C for 30 s. Normalization was performed with PPIA, GAPDH, and / or SRSF4 as internal reference controls as indicated. Data were analyzed using StepOne Software v2.3. Primer sequences used in this study are provided in Supplementary Data 4.

### Protein isolation, protein quantification, and western blot

Cells were briefly washed with 1× PBS and lysed in 200 µl RIPA buffer (50 mM Tris-HCl pH 7.5, 150 mM NaCl, 1% Triton X-100, 0.5% Na-Deoxycholate, 0.1% SDS, 1% DTT) supplemented with 1× Protease (Roche, 4693132001) and phosphatase inhibitors (Sigma-Aldrich, 4906837001) on ice for 30 min with intermittent mixing. The lysate was centrifuged at 17,000 *g* for 10 min at 4 °C for clearing. The supernatant was transferred to new tubes and flash-frozen until further analysis. Protein quantification was performed using the Pierce BCA Protein Assay Kit (Thermo Fischer, 23224) as per the manufacturer’s instruction. Samples were boiled for 10 min at 95 °C in 4× Laemmli Buffer (0.25 M Tris pH 6.8, 20% glycerol, 10% SDS, 355 mM 2-mercaptoethanol, 0.002% bromophenol blue) before loading onto 10% SDS-PAGE gels. Proteins were transferred to nitrocellulose membranes using transfer buffer (0.25 M Tris-base, 1.92 M Glycine, 1% SDS with 20% methanol) at 120 V for 90 min. The membrane was blocked with 5% milk in 1× TBS-T (247 mM Tris, 1.37 M NaCl, 26.8 mM KCl, 0.05% Tween-20) for 30 min. Blots were incubated with the primary antibodies in 5% milk in 1× TBT-T overnight at 4 °C with indicated dilutions. After incubation with primary antibody, blots were washed five times with 1× TBS-T for 5 min at room temperature on a shaker. Next, blots were incubated for 1 h at room temperature with respective anti-mouse or anti-rabbit HRP-conjugated secondary antibodies (Jackson ImmunoResearch Laboratories, 115-035-003 and 111-035-144) were used at a dilution of 1:2500. After incubation with secondary antibody, blots were washed again for five times with 1× TBS-T for 5 min at room temperature on a shaker. Membranes were developed using Supersignal Pico (Fisher Scientific, 34580). Images were acquired on the Intas ChemoCam Imager, and signal quantification was performed using LabImage 1D software. A list of antibodies and dilutions used is provided in Supplementary Data 5.

### Cell viability

CellTiter-Glo Luminescent Cell Viability Assay (Promega kit, G7572) was performed 72 h after knockdown with the respective siPOOLs. At the time point for the assay, growth medium was removed from the cells using multi-channel pipette and 60 µL of 1:4 CellTiter-Glo reagent: 1× PBS was added to the cells. Plate was incubated for 15 min at room temperature in the dark using an orbital shaker. After the incubation, chemiluminescence was measure using luminometer (Fluoroskan Ascent FL, Thermo Scientific). Data obtained were normalized to siPOOL-negative control.

### Rapid amplification of cDNA ends (RACE)

The total RNA (treated with DNase I) from HLE cells was used for first-strand cDNA synthesis. The SMARTer RACE cDNA Amplification Kit (Clontech, 634923) was used to perform 5′- and 3′-RACE analysis according to the manufacturer’s instructions. The gene-specific primers used for RACE are listed in Supplementary Data 6.

### Subcellular fractionation

Subcellular fractionation was performed in HLE cells to separate chromatin-associated, nucleoplasmic and cytoplasmic fractions as previously described^71^. Fraction-specific controls were used to assess the quality of fractions obtained (chromatin fraction: *NEAT1*, *MALAT1*; nucleoplasmic fraction: *RNU-1*; cytoplasmic fraction: *DANCR*). Primer sequences are provided in Supplementary Data 4.

### Cell proliferation

BrdU incorporation efficiency of cells was measured at 72 h post knockdown with respective siPOOLs using the Cell Proliferation Assay kit (Roche, 11669915001) as per the manufacturer’s instructions.

### Cell cycle analysis

Seventy-two hours post treatment with siPOOLs, the cells were trypsinized and fixed in 70% ethanol overnight at −20 °C. Fixed cells were pelleted and washed with 1× PBS. After washing, cells were resuspended in 1× PBS containing 100 µg/ml RNase A (Sigma, 10109169001) and incubated at 37 °C for 30 min. Post RNase treatment, the cells were stained with 100 µg/ml propidium iodide (Sigma-Aldrich, P4170). In total, 10,000 cells were acquired on BD FACSCanto II Flow Cytometer, and data analysis was performed using FlowJo v10 software.

Triapine (Selleckchem, S7470), the RRM2 inhibitor^45^, was dissolved in DMSO, and HLE cells were treated with respective concentrations. Cell cycle analysis was performed as described, and data were analyzed using FlowJo software.

### Apoptosis Caspase-Glo assay

The assay was performed using the Caspase-Glo assay kit from Promega (G8091) in a 96-well format. Cells were transfected with 10 nM of respective siPOOLs and incubated at 37 °C with 5% CO_2_ for 72 h. The supernatant was discarded and replaced with diluted (1:2 in 1× PBS) Caspase-Glo solution. The plate was incubated for 60 min at RT in the dark. Luminescence was measured with a FLUOstar Omega microplate reader (BMG Labtech).

### Senescence-associated β-Gal assay

The Cells were reverse transfected with the respective siPOOLs on a six-well plate, and SA-β-Gal activity was detected 96 h post transfection. Cells were washed with 1× PBS and fixed at room temperature with 0.5% glutaraldehyde for 20 min. Cells were washed twice with 1× PBS supplemented with 1 mM MgCl_2_ (pH 6.0) for 10 min on a rocker. In all, 2 ml X-Gal staining solution (1× PBS containing 1 mM MgCl_2_, 41 mg of potassium hexacyanoferrate (III), 52.5 mg of potassium hexacyanoferrate (II) trihydrate, 1 mg/ml X-Gal (5-bromo-4-chloro-3-indolyl-beta-D-galacto-pyranoside), pH 6.0) was added, and the dishes were sealed with parafilm and incubated overnight at 37 °C. Next day, the cells were washed three times with distilled water, and microscopy pictures were taken using a 10x objective of a Zeiss Cell Observer microscope. For analysis, 100 cells were counted, and percent senescent cells per condition are depicted in the bar graph.

### Chick chorioallantoic membrane (CAM) assay

Pathogen-free fertilized eggs were purchased from Valo Biomedia GmbH and incubated in an incubation oven with 60% humidity at 37 °C. On day 8, *lincNMR* was knocked down with 10 nM siPOOLs using a forward transfection protocol described before in HLE cells. On day 9, eggs were windowed with an electric drilling tool. On the same day, in parallel, 1 × 10^6^ transfected HLE cells were resuspended in 10 µL growth media, mixed with 10 µL Matrigel (Corning, 354262) and incubated for 10 min at 37 °C and 5% CO_2_. The cell-matrigel mix was seeded onto the CAM, and the window was sealed for five days and incubated at 37 °C with 60% humidity. During these days, eggs were observed, and dead or injured embryos were excluded from further experiments. On day 6, after seeding the cells on the CAM, chicken embryos were euthanized by a quick decapitation, and tumors were harvested from the CAM. Harvested tumors were processed, cleaned, and collected in ice-cold 1× PBS. Images of tumors from HLE cells transfected with negative control siPOOL, and two independent siPOOLs targeting *lincNMR* were taken. Tumors were weighed on a microscale.

### Colony formation assay

Colony formation assays were performed as described in ref. ^72^. In brief, HLE cells were reverse transfected with 10 nM of respective siPOOLs. Twenty-four hours later, 500 cells were reseeded into six-well plates to allow formation of colonies for 14 days. After the incubation, cells were fixed with 6% glutaraldehyde and stained with 1% crystal violet solution. After subsequent washes, plates were allowed to dry at room temperature, and colonies were counted.

### In vivo RNA antisense purification (in vivo RAP-MS)

Biotinylated DNA oligos complementary to *lincNMR* sequence were ordered from IDT, sequences are available in Supplementary Data 7. One billion HLE cells were used per pulldown per condition per biological replicate. In vivo RAP-MS was performed as per the protocol described previously^41^.

### Generation of plasmids and mutagenesis

Gateway entry vectors were obtained from the DKFZ plasmids and clone repository. *LincNMR-001* was amplified using the primers listed in Supplementary Data 10. Gateway LR reaction was performed with 50–150 ng of entry vector using LR Clonase II (Thermo, 11791020) as per the manufacturer’s instructions into the gateway destination vector pFRT-Flag/HA. Mach1 cells were used for transformation. Mini-Prep was performed using the NucleoSpin^®^ Plasmid kit (Macherey & Nagel, 740588.250). Midi-Prep was done using the PureLink™ HiPure Plasmid kit (Invitrogen, K210004). Services from Eurofins genomics/GATC were used for sequencing with CMV.for CGCAAATGGGCGGTAGGCGTG and BGH.rev TAGAAGGCACAGTCGAGG primers. Finally, cells were transfected with respective plasmid and empty vector pFRT-Flag-HA-ΔCmR-ΔccdB as a control plasmid. Overexpression was confirmed by western blot using anti-Flag-M2 or anti-HA antibody (Supplementary Data 5).

Mutagenesis of the predicted high-confidence YBX1-binding sites (RBPmap) in the *lincNMR* transcript was performed using Phusion DNA polymerase as per supplier’s instructions (NEB, M0530S). Primers used for performing PCR are listed in Supplementary Data 10.

### UV cross linking RNA immunoprecipitation (UV–RIP) assay

Cell Seeding, Transfection, UV Cross linking, harvesting: on day 1, 4 × 10^6^ HLE cells were seeded onto a 15-cm dish. On day 2, 2 µg of the respective constructs (Empty vector plasmid pFRT-Flag-HA-ΔCmR-ΔccdB or pFRT-Flag/HA-YBX1) were transfected using TurboFect transfection reagent with a forward transfection protocol. On day 4, cells were UV cross linked at a wavelength of 254 nm using 0.8 J/cm^2^ (instrument setting: 8000 × 100 µJ/cm^2^) and then lysed in high-strength cell lysis buffer (10 mM Tris-HCl pH 7.5, 500 mM lithium chloride, 0.5% dodecyl maltoside, 0.2% SDS, 0.1% sodium deoxycholate) supplemented with inhibitors cocktail containing SUPERase In (Thermo Scientific, AM2696), protease and phosphatase inhibitors. Lysate was passed through a syringe to break up the pellet, and DNase digestion was performed as per the manufacturer’s instruction using the Turbo DNA-free Kit (Ambion, AM1907). Protein quantification was performed using the BCA reagent, and overexpression of YBX1 was confirmed by probing with anti-HA antibody on a western blot.

Pulldown: 1.5 µg of cell lysate was used to perform UV– RIP using anti-Flag magnetic beads, and IP was confirmed by probing for anti-HA using western blot. For UV–RIP, 150 µl of anti-Flag magnetic beads were used per pulldown. Beads were prewashed 5× using 1× TBS and resuspended in supplemented cell lysis buffer. In total, 5% lysate was removed for the Input fraction, and 150 µl of prewashed beads were added to the cell lysate and incubated for 1 h at 4 °C with rotation. After the incubation, beads were magnetically separated. 50 µl of flow-through was saved to confirm the depletion of YBX1, and the remaining flow-through was discarded. Beads were washed 2× with low salt-wash buffer (0.1% SDS, 0.5% sodium deoxycholate, 0.5% NP-40, 0.01 M NaCl, 0.002 M KCl, 0.001 M Na_2_HPO_4_, 0.0001 M KH_2_PO_4_) and then with high salt-wash buffer (0.1% SDS, 0.5% sodium deoxycholate, 0.5% NP-40, 0.05 M NaCl, 0.010 M KCl, 0.005 M Na_2_HPO_4_, 0.0005 M KH_2_PO_4_) with rotation for 5 min per wash. Beads were magnetically separated and resuspended in 500 µl of RNase-free pure water. Beads were then separated into 20% (for protein extraction) and 80% (RNA isolation) to confirm the IP and *lincNMR* pulldown, respectively. Antibodies and magnetic beads used are listed in Supplementary Data 5.

Elution of RNA: RNA was eluted from the beads by reversal of UV cross linking in high salt buffer combined with Proteinase K digestion (160 µl of Proteinase K buffer (125 mM Tris-HCl pH 7.8, 62.5 mM NaCl, 12.5 mM EDTA) and 40 µl of Proteinase K) by incubating at 37 °C with shaking for 30 min at 1000 rpm. Next, after addition of 500 µl of Trizol to the tube, the tube was vortexed for 10 s and stored at −20 °C until ready for RNA isolation. RNA isolation, reverse transcription, and qPCR were performed as described above.

Elution of proteins: Captured, washed beads were boiled with 1× SDS loading buffer at 95 °C for 10 min, and western blot was performed with input, supernatant, and flow-through samples to confirm the YBX1 pulldown.

### In vitro *lincNMR* RNA-affinity purification

In vitro *lincNMR* RNA-affinity purification was performed as described previously^73^. The *lincNMR* sequence (1100 nt) was cloned into a pcDNA3.1 vector, which was further linearized using EcoRV and MluI digestion. As a negative control, the lncRNA *HULC* (560 nt) was used for comparison, which had been previously cloned^73^. The MEGAscript T7 Transcription Kit (Ambion, AMB13345) was used according to the manufacturer’s instructions for in vitro transcription. In vitro transcribed biotinylated lncRNAs were used to pull down the interacting proteins from HLE cell lysate using streptavidin sepharose beads (GE Healthcare, 17-5113-01). After subsequent washing steps, proteins were eluted using wash buffer (20 mM HEPES pH 7.9, 300 mM KCl, 10 mM MgCl_2_, 0.01% NP-40, 1 mM DTT) supplemented with 50 μg/ml RNase A. Eluates were acetone precipitated and the pellet was washed twice with 80% ethanol. The washed pellet was dissolved in 2× SDS sample by heating at 95 °C for 5 min. Western blot was performed to identify the RNA interacting proteins.

### Triple-label SILAC-MS

Incorporation of Light (Lys0, Arg0), Medium (Lys4, Arg6), and Heavy (Lys8, Arg10) Isotopic Labels: The SILAC Protein Quantitation Kit RPMI-1640 (Life technologies, 8992) was used to generate light- and heavy-labeled HLE cells. For generation of medium-labeled cells, 4,4,5,5-D4 L-Lysine-2HCl (Fischer Scientific, 11305402) and L-Arginine-HCl ^13^C_6_ (Life technologies, 88210) were individually purchased. 4,4,5,5-D4 L- Lysine-2HCl and L-Arginine-HCl ^13^C_6_ were mixed to achieve a final concentration of 0.46 mM and 0.47 mM, respectively, in SILAC RPMI-1640 medium (Life Technologies, A2494401). Cells were grown in the respective medium supplemented with 200 µg/ml L-Proline (Thermo Fischer Scientific, 88211) for at least 8–10 passages in a humidified incubator at 37 °C with 5% CO_2_ to achieve >99% incorporation of the respective labels as verified by mass spectrometry.

Cell culture, transfections, lysate preparation, protein quantification, LC-MS/MS: After confirmation of label incorporation, isotopically labeled HLE cells were grown to 80% confluency. Cells were reverse transfected with the respective siPOOLs targeting *lincNMR* or the control siPOOL. Lysates were harvested 48 h post transfection. Cells were lysed in 200 µl RIPA buffer (50 mM Tris-HCl pH 7.5, 150 mM NaCl, 1% Triton X-100, 0.5% Na-deoxycholate, 0.1% SDS, 1% DTT) supplemented with protease and phosphatase inhibitors. In addition, 0.1% benzonase (Merck, Darmstadt, Germany) was added to digest nucleic acids. After incubation on ice for 1 h, cell lysates were cleared by centrifuging at 15,000 *g*, for 30 min at 4 °C. The 2D Quant Kit (GE Healthcare, 806483356) was used to measure protein concentrations.

### YBX1 ChIP assay

Chromatin immunoprecipitation (ChIP) assay was performed as described before^74^ to identify YBX1 interacting promoter regions. HLE cell lysates were used for the ChIP experiment using YBX1 antibody (Abcam, ab12148) and isotype control rabbit IgG antibody (Abcam, ab171870). ChIP pull-down DNA was analyzed using the qPCR and primers listed in Supplementary Data 8.

### Dual-luciferase reporter assay

HLE and FLC-4 cells were reverse transfected with 10 nM of control siPOOL and siPOOLs targeting *lincNMR* on day 1. Control pRL-TK/pRL-SV40 reporter constructs and Y-Box-TATA-Luc were co-transfected using a forward transfection protocol on day 2. Cell culture medium was changed on day 3. Luciferase reporter assay was performed using a Dual-Luciferase Reporter Assay System kit (Promega, E1960) on day 4 as per the manufacturer’s instruction, and luminescence was measured on Spectra Max M5e (Molecular Devices).

For the characterization of the YBX1 impact on RRM2, TK1, and TYMS promoter regions, genomic DNA from HLE cells was PCR-amplified using Phusion polymerase (NEB, M0530). The first round of PCR was performed using primers without restriction sites. After gel elution of PCR purified products, a nested PCR was performed using primers with restriction sites. Gel purified PCR products and empty pcDNA3.1 vector were digested using Xhol / NotI and ligation was performed using T4 DNA ligase (Thermo, EL0011). Primers used for creating the constructs are listed in Supplementary Data 9.

### dNTP quantification

Cellular dNTPs were extracted from cells (HLE and FLC-4) transfected with the respective siPOOLs 72 h post transfection as per a previously published protocol^75^. The dried dNTPs were resuspended to proper volumes of water and added to the HIV-1 RT-mediated single dNTP incorporation reactions. The percent of primer extension were converted to the incorporated dNTP amounts, and the determined dNTP amounts were normalized by 1 million cells for comparison. Further, data were normalized to negative control siPOOLs.

### dNTP bathing and rescue assay

In total, 1250 cells were reverse transfected with the respective siPOOLs in a clear bottom 96-well plate. The desired concentration of extracellular dNTPs was added to the cells 24 h later. A cell proliferation assay was performed at 96 h post siPOOL transfection to assess the proliferation of the cells.

### RNA copy number detection

1 µg of RNA was used for reverse transcription with MaximaRT (Thermo Fischer EP0751) from respective cell lines (HLE, HLF, SNU-387 and FLC-4). 1:10 diluted cDNA was used for qPCR. Primers used for qPCR are listed in Supplementary Data 4. Plasmids containing *lincNMR*-001 sequence were linearized using BstBI digestion and gel purified. Serial dilutions were made in the range of 0.02 pg – 5 pg. The copy number was calculated using the online tool https://cels.uri.edu/gsc/cndna.html.

### Statistics and reproducibility

All experiments were performed at least in three biological replicates, and information about statistical tests used is detailed in the respective figure legends. Exact *P*-values are provided in the Source Data file. Numbers of replicates always refer to independent biological replicates.

### Reporting summary

Further information on research design is available in the Nature Research Reporting Summary linked to this article.

## Supplementary information

Supplementary Information

Description of Additional Supplementary Files

Supplementary Data 1

Supplementary Data 2

Supplementary Data 3

Supplementary Data 4

Supplementary Data 5

Supplementary Data 6

Supplementary Data 7

Supplementary Data 8

Supplementary Data 9

Supplementary Data 10

Reporting Summary

## Data Availability

Sequencing data from 5′- and 3′-RACE experiment supporting both *lincNMR* isoforms have been deposited at Genbank with accession numbers MK652436 and MK652437. Source data for figures shown in this study are available upon request if not available in Supplementary Data and in the attached source data files. The source data underlying Fig. 4c, e and Supplementary Figs. 4e, f, 2i as well as Figs. 1a–d, f, g, 2a, b, d, 3c–g, 4d, f, 5a, b, d, f, 6d, e, 7a–d, f, and Supplementary Figs. 1e, f, 2a–h, 3a–d, 4c, g, h, 5b–e, 6a–c are provided as Source Data files.

## Source data

Source Data
