# Supplementary material for: The lncRNA lincNMR regulates nucleotide metabolism via a YBX1 - RRM2 axis in cancer
Source: Nat Commun. 2020 Jun 25;11:3214. doi: 10.1038/s41467-020-17007-9 (PMC7316977; doi:10.1038/s41467-020-17007-9)
Supplement: Supplementary file 1 — Supplementary Information [file 41467_2020_17007_MOESM1_ESM.pdf]

The lncRNA *lincNMR* regulates Nucleotide Metabolism via a YBX1 - RRM2 Axis in Cancer

Minakshi Gandhi, Matthias Groß, Jessica M. Holler, Si'Ana A. Coggins, Nitin Patil, Joerg H. Leupold, Mathias Munschauer, Monica Schenone, Christina R. Hartigan, Heike Allgayer, Baek Kim, Sven Diederichs

Supplementary Information

Supplementary Figure 1

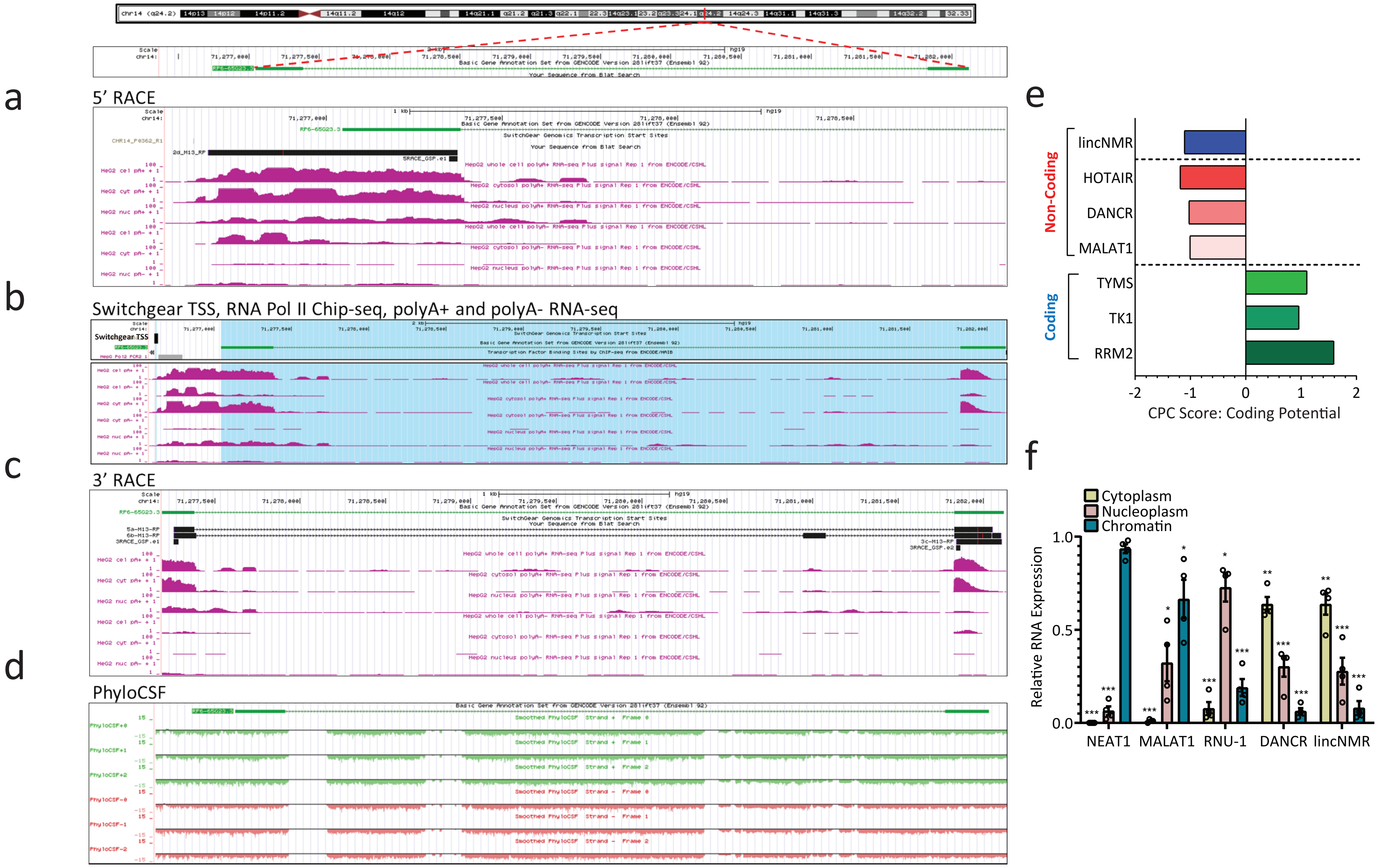

Supplementary Figure 1

Characterization of *lincNMR*: Gene boundaries, sequence, coding potential and subcellular localization.

a. *lincNMR* genomic orientation from UCSC genome browser derived from comprehensive gene annotation set GENCODE tracks. Black bar depicts the blat sequence search representing 5' RACE data. Additionally, subcellular fraction-specific HepG2 RNA-Seq data from ENCODE/CSHL tracks confirming the further upstream transcription start site of the *lincNMR* transcript and predominant cytoplasmic localization of *lincNMR*. Gene-specific primer 5RACE_GSP.e1 was used for amplification.
b. UCSC genome browser tracks depicting data from Switchgear transcription start site (TSS) annotation supporting longer *lincNMR* exon with another TSS. Also shown is grey bar depicting RNA Pol2 binding site from Transcription factor binding site ChiP-Seq data set from ENCODE/HAIB.
c. *lincNMR* genomic orientation from UCSC genome browser derived from comprehensive gene annotation set GENCODE tracks. Black bar depicts the blat sequence search representing 3' RACE data. Two different gene specific primers (GSP) were used for 3'RACE, 3RACE_GSP.e1 and 3RACE_GSP.e2.
d. PhyloCSF tracks indicates the lack of coding potential of *lincNMR* in all three translation frames.
e. Coding Potential Calculator: Bar graph representing coding potential scores for a set of coding (RRM2, TK1 and TYMS) and non-coding (*lincNMR*, HOTAIR, DANCR and MALAT1) genes where positive scores indicate a gene coding for proteins and negative scores confirm the non-coding potential of lncRNAs.
f. Subcellular fractionation of HLE cells and subsequent RT-qPCR confirms the cytoplasmic and partially nucleoplasmic localization of *lincNMR*. Fraction-specific controls are included to ensure fractionation quality (Chromatin: NEAT1, MALAT1; Nucleoplasmic: RNU1; Cytoplasmic: DANCR). Data represent mean and error bars represent SEM (n=4). Significance was calculated by unpaired, two-tailed t-test with *, $P<0.05$; **, $P<0.01$; ***, $P<0.001$.

Supplementary Figure 2

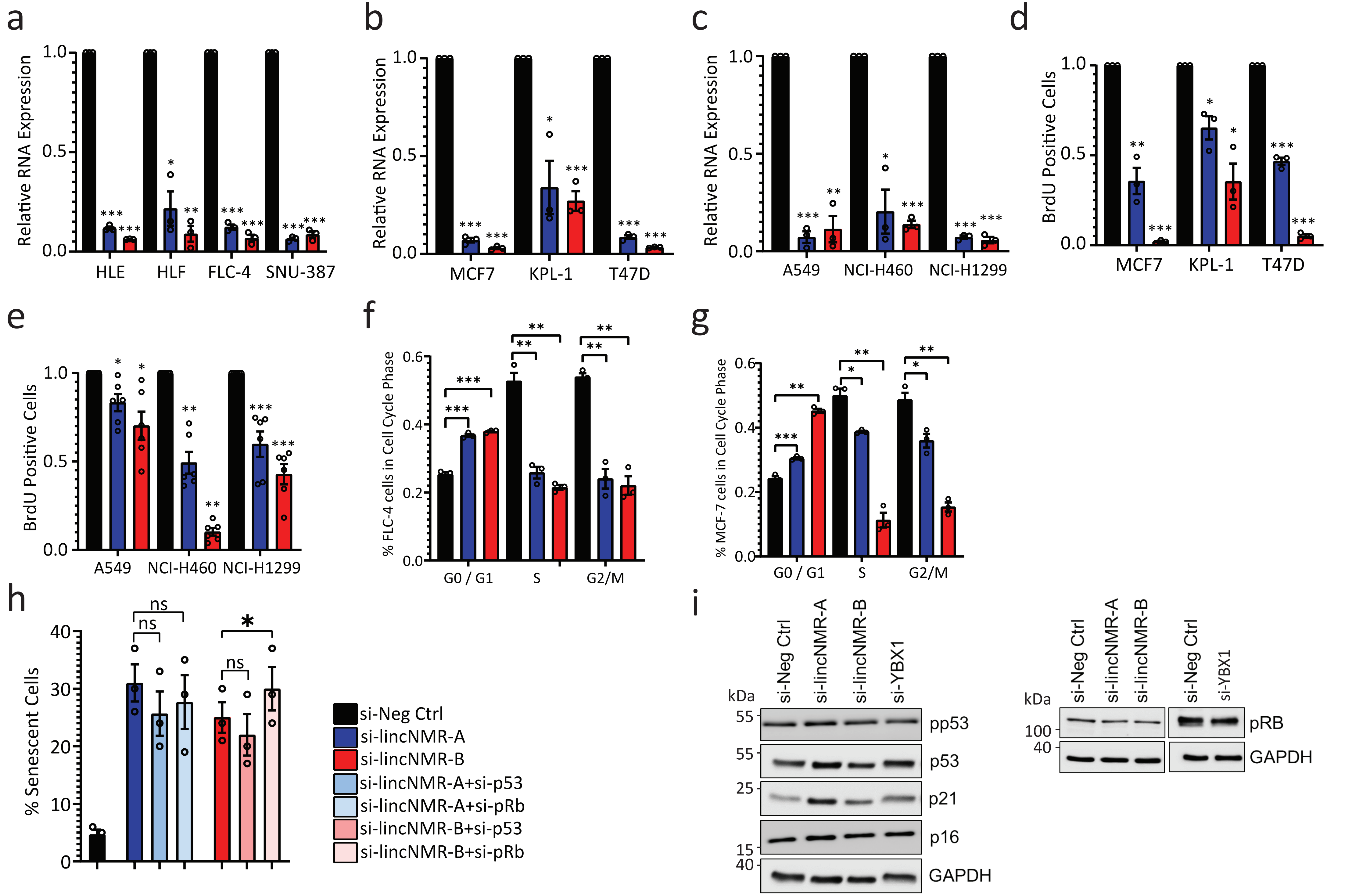

Supplementary Figure 2

Depletion of *lincNMR* leads to impaired cell proliferation and G1 arrest in multiple cancer entities

a-c. *LincNMR* knockdown with 10 nM of two independent siPOOLs (si-*lincNMR*-A and si-*lincNMR*-B) leads to (a) 80 – 94 % silencing in multiple liver cancer cell lines (HLE, HLF, FLC4, SNU-387; 24h), (b) 75 – 99 % silencing in multiple breast cancer cell lines (MCF-7, KPL-1, T47D; 72h) and (c) 80 – 93 % silencing in multiple lung cancer cell lines (A549, NCI-H460, NCI-H1299; 72h).

d-e. Depletion of *lincNMR* with 10 nM siPOOLs invokes a proliferation decrease in (d) multiple breast cancer cell lines (MCF7, KPL-1, T47D; 72h) and (e) in multiple lung cancer cell lines (A549, NCI-H460, NCI-H1299; 72h).

f-g. Silencing of *lincNMR* with 10 nM siPOOLs induces cell cycle arrest in G0/G1 phase at 72 h post transfection in (f) FLC-4 cells and (g) MCF-7 cells.

h. SA-β -Gal assay quantification represented in bar graph as % senescent cells after silencing of *lincNMR* and with or without co-depletion of *TP53* (p53) or *RB1* (pRb) in HLE cells using 10 nM siPOOLs at 96 h post transfection.

i. Representative western blots: phospho-p53 (pp53), p53, p21, p16, pRB and GAPDH after depletion of *lincNMR* (lincNMR-A and lincNMR-B) or YBX1 in HLE cells (n=6). si-NegCtrl was used as a negative control siPOOL whereas GAPDH was used as a loading control for western blot. Antibodies and their respective dilutions used are listed in Supplementary Data 5.

a-c: *LincNMR* was measured using RT-qPCR and data normalized to *SRSF4* and negative control siPOOL.

f, g: Analysis was performed using Cell Cycle Platform in FlowJo v10 software.

a-h: Data represent mean (n=3) and error bars represent SEM. Significance was calculated by unpaired, two-tailed t-test with *, $P<0.05$; **, $P<0.01$; ***, $P<0.001$.

Supplementary Figure 3

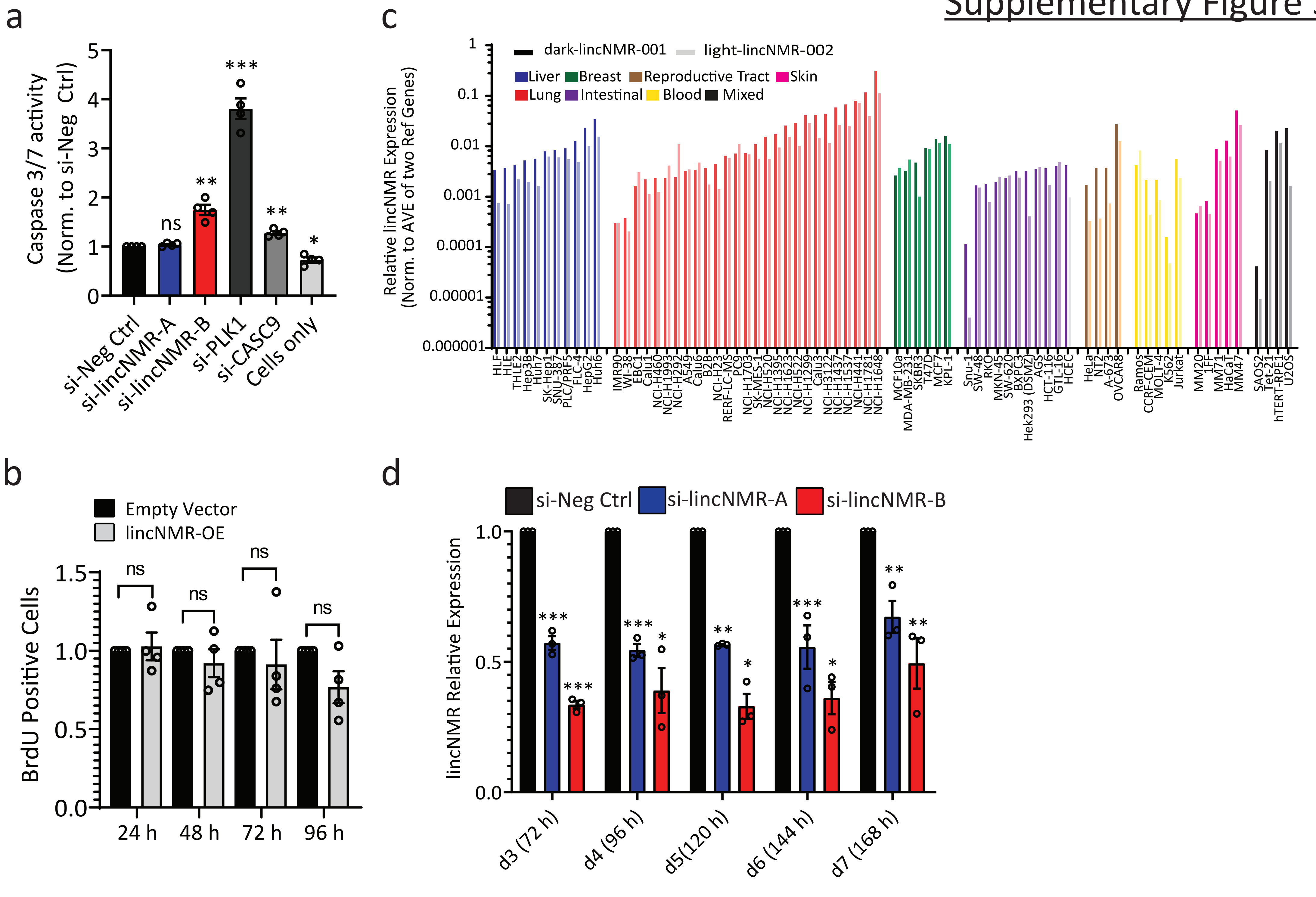

Supplementary Figure 3

LincNMR is expressed in many cancer cell lines

a. Caspase-Glo 3/7 assay measuring induction of apoptosis at 72 h after depletion of *lincNMR* using 10 nM siPOOLs in HLE cells with siPOOLs targeting PLK1 and CASC9 used for comparison (n=3). Data represent mean and error bars represent SEM. Significance was calculated by unpaired, two-tailed t-test with *, $P<0.05$; **, $P<0.01$; ***, $P<0.001$.

b. Cell proliferation assay at multiple time points (24 h, 48 h, 72 h and 96 h) after transfecting cells with plasmids overexpressing *lincNMR* in HLE cells with empty vector used as a negative control plasmid (n=3). Data represent mean and error bars represent SEM. Significance was calculated by unpaired, two-tailed t-test with *, $P<0.05$; **, $P<0.01$; ***, $P<0.001$.

c. RT-qPCR data in a panel of 73 human cell lines showing expression of the two *lincNMR* isoforms (*lincNMR*-amp#1, *lincNMR*-amp#2) across multiple cancer types (liver, lung, breast, skin, intestinal, reproductive tract, blood etc.). The cell line panel also includes non-cancer cell lines (IMR90, WI-38 and MCF10a). Expression data shown is normalized to average of two reference genes SRSF4 and PPIA.

d. Time course of knockdown efficiency determined by RT-qPCR to verify stable knockdown of *lincNMR* until seven days after transfection with 10 nM siPOOLs in HLE cells. Data was normalized to SRSF4 and negative control siPOOL. Data represent mean and error bars represent SEM (n=3). Significance was calculated by unpaired, two-tailed t-test with *, $P<0.05$; **, $P<0.01$; ***, $P<0.001$.

Supplementary Figure 4

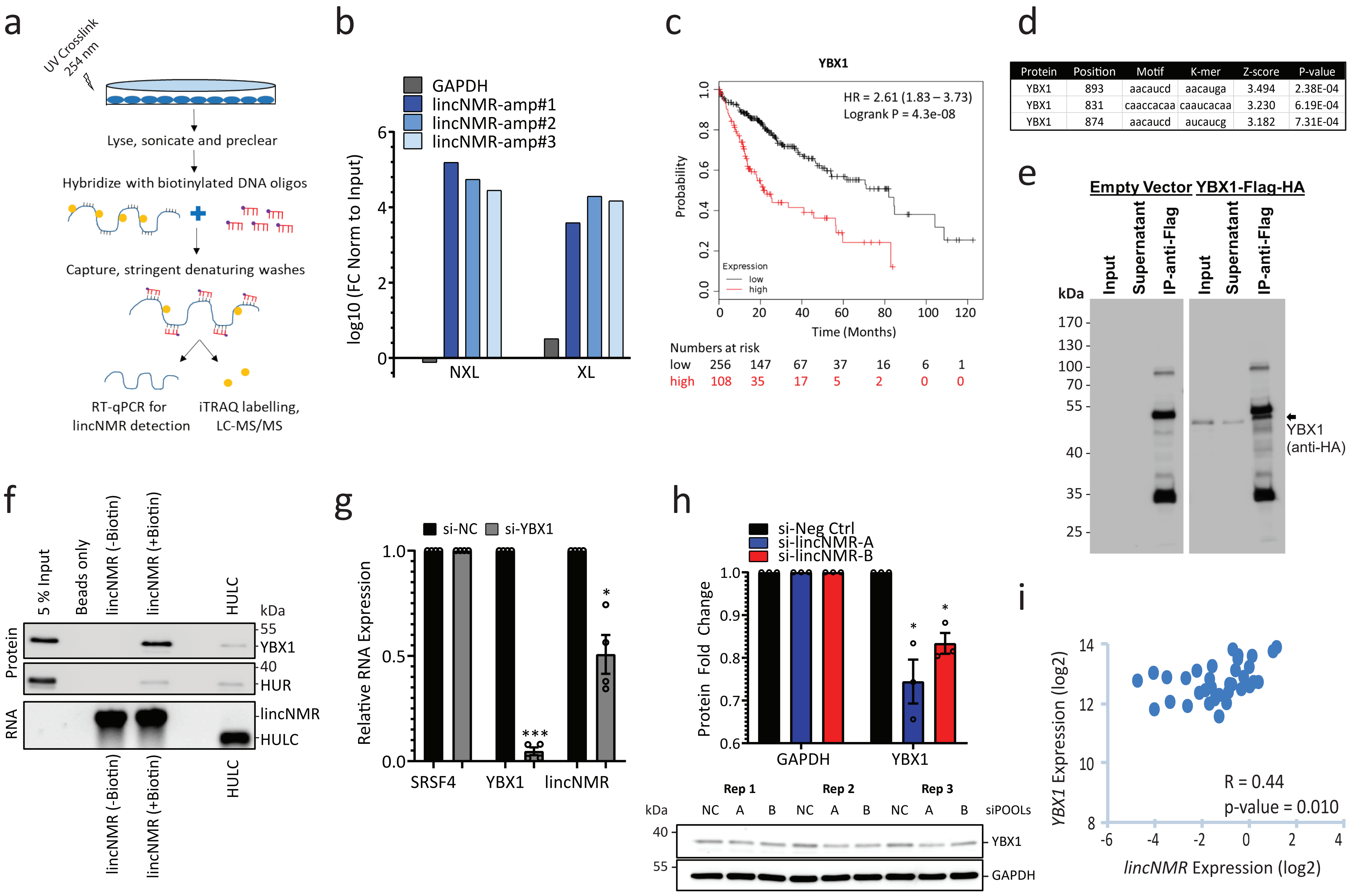

Supplementary Figure 4

In vivo RNA Antisense Purification (RAP-MS) and knockdown of YBX1

a. Schematic overview of RAP-MS used for identification of proteins interacting with *lincNMR*.

b. *lincNMR* RAP-MS pulldown efficiency: data represent log10 fold change normalized to reference gene *PPIA* and input in HLE cells with three different *lincNMR* amplicons and *GAPDH* as additional negative control (n=2). NXL: no crosslink, XL: crosslink.

c. Kaplan-Meier plot correlating high *YBX1* expression to poor survival in hepatocellular carcinoma patients (TCGA data, KM plotter), log-rank test.

d. YBX1 binding sites in *lincNMR* transcript as predicted by RBPmap and ranked using Z-score and Fisher's exact test.

e. Representative UV-RIP western blot: validation of the pulldown of exogenous YBX1 tagged with Flag and HA. Anti-Flag antibody magnetic beads were used for pulldown and anti-HA antibody was used for western blot in HLE cells (n=4). Input, supernatant and IP fractions on 10% SDS-PAGE gel. Empty vector served as control.

f. Top panel: Representative western blot of *in vitro* RNA Affinity Purification validating the interaction of biotinylated *lincNMR* with endogenous YBX1 (HLE lysate). Non-biotinylated *lincNMR,* beads, the lncRNA *HULC* or the RBP HUR served as negative controls (n=3). Bottom panel: Agarose gel electrophoresis of *in vitro* transcribed RNA showing successful transcription of non-biotinylated and biotinylated *lincNMR* and biotinylated *HULC* (n=3).

g. Depletion of YBX1 (10 nM siPOOLs; 72h) in HLE cells was effective and lead to downregulation of *lincNMR* expression. *LincNMR* and *YBX1* RNA were measured by RT-qPCR and normalized to *SRSF4* and negative control siPOOL.

h. Depletion of *lincNMR* (10 nM siPOOLs; 72h) in HLE cells lead to marginal, but significant downregulation of YBX1 protein. YBX1 protein fold change was normalized to the loading control GAPDH and negative control siPOOL. Below, western blot image is shown.

i. *LincNMR* expression levels significantly correlate with *YBX1* mRNA expression (R=0.44) in hepatocellular carcinoma patients (TCGA through TANRIC platform) [32].

g-h: Data represent mean and error bars represent SEM (n=3). Significance was calculated by unpaired, two-tailed t-test with *, $P<0.05$; **, $P<0.01$; ***, $P<0.001$.

Supplementary Figure 5

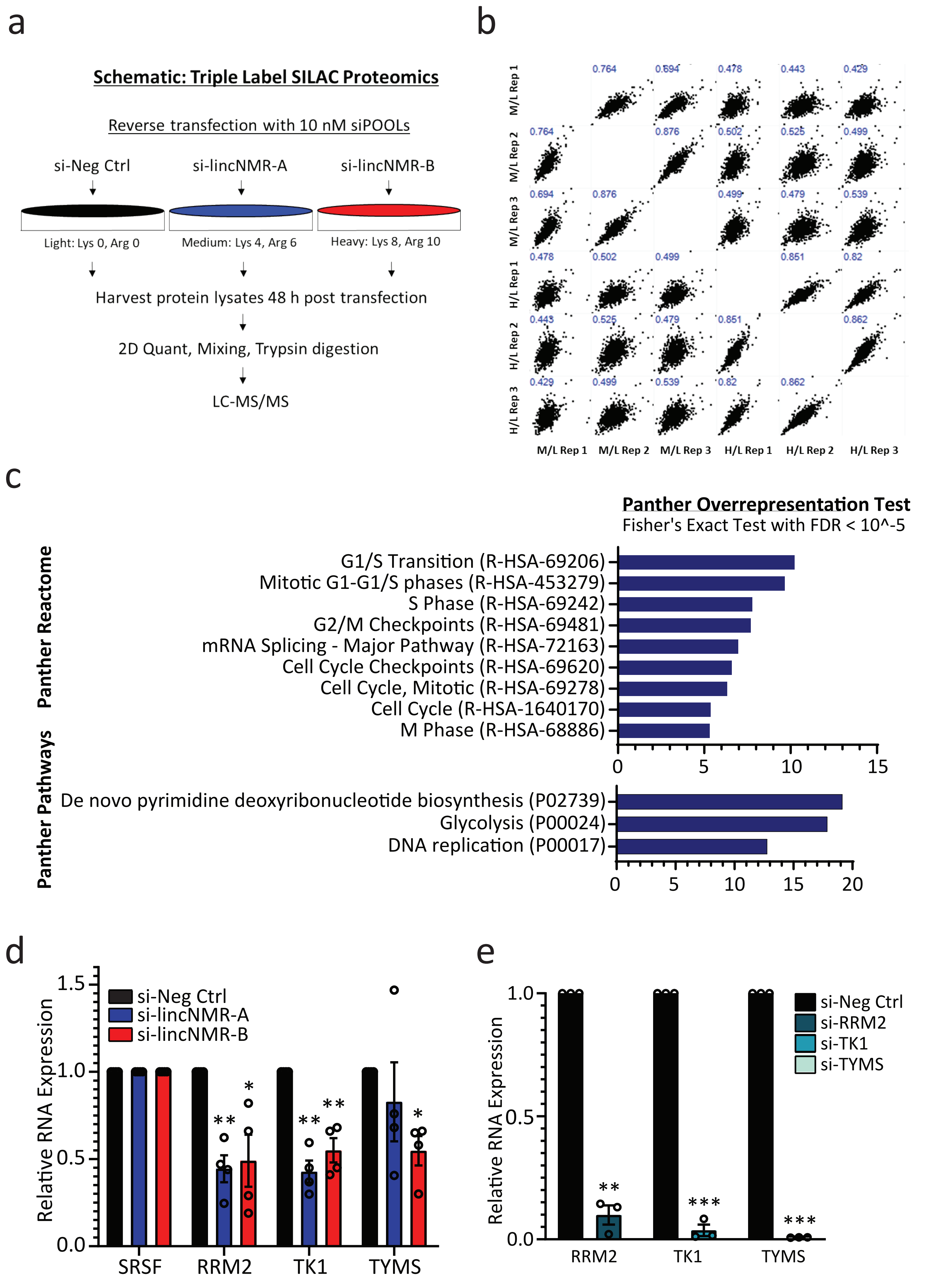

Supplementary Figure 5

Triple-label SILAC-MS: Schematic, reproducibility and gene ontology analysis of deregulated protein candidates

a. Schematic outline of triple-label SILAC-MS experiment to identify proteins deregulated upon *lincNMR* silencing.
b. Scatter plots depicting all deregulated proteins identified within and across three biological replicates using two independent siPOOLs (si-lincNMR-A: Medium (M), si-lincNMR-B: Heavy (H), vs. control siPOOL: Light (L)) in triple-label SILAC-MS. Cell lysates were harvested 48 h after depletion of *lincNMR* with 10 nM siPOOLs. Data represents fold change normalized to negative control siPOOLs. A total of 2088 proteins were identified across biological replicates and only candidate deregulated with both siPOOLs were selected for further analysis. Pearson correlation coefficient is displayed on the scatter plots.
c. Panther overrepresentation test: Fisher's exact test was performed with FDR calculation for multiple testing correction on a dataset of 242 deregulated candidates ($P<0.001$) obtained from triple-label SILAC-MS experiment after depletion of *lincNMR* in HLE cells. Depicted is the fold enrichment of key terms associated with these deregulated hits from SILAC-MS in the PANTHER Reactome and PANTHER Pathways.
d. Depletion of *lincNMR* with two independent siPOOLs (si-lincNMR-A and si-lincNMR-B) leads to downregulation of *RRM2*, *TK1* and *TYMS* RNA in HLE cells at 72 h after *lincNMR* knockdown with 10 nM siPOOLs (n=3).
e. RT-qPCR data showing knockdown efficiency of RRM2, TK1 and TYMS in HLE cells with 10 nM respective siPOOLs at 72 h post knockdown (n=3).

d, e: Data shown is normalized to negative control siPOOL and reference gene SRSF4. Data represents mean and error bars represent SEM. Significance was calculated by unpaired, two-tailed t-test with *, $P<0.05$; **, $P<0.01$; ***, $P<0.001$.

Supplementary Figure 6

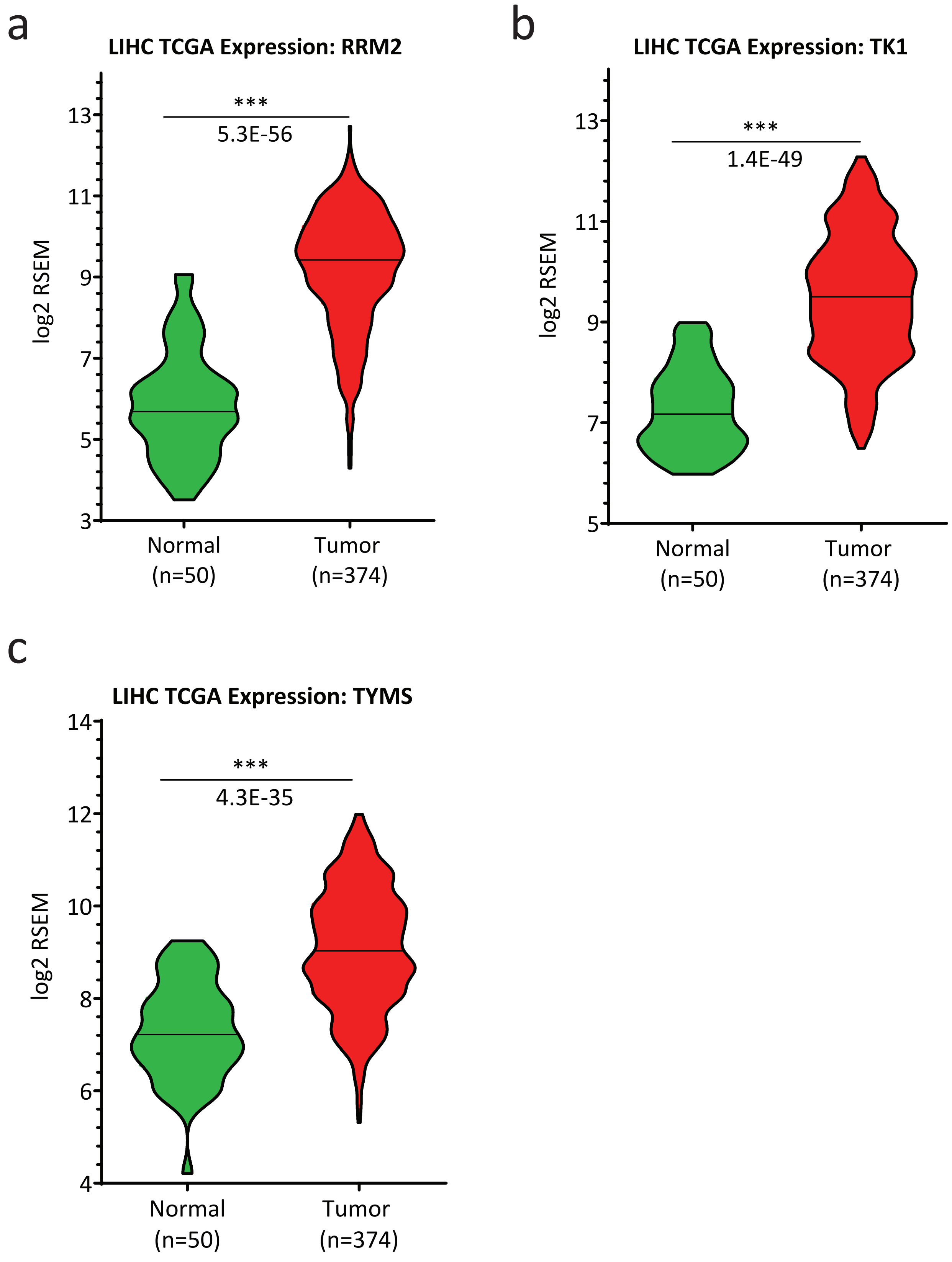

Supplementary Figure 6

RRM2, *TK1* and *TYMS* are expressed and induced in hepatocellular carcinoma

a. *RRM2* is expressed and regulated in liver cancer (N=normal, T=tumor; data obtained from TCGA data portal). LIHC=Hepatocellular Carcinoma (N=50, T=394).
b. *TK1* is expressed and regulated in liver cancer (N=normal, T=tumor; data obtained from TCGA data portal). LIHC=Hepatocellular Carcinoma (N=50, T=394).
c. *TYMS* is expressed and regulated in liver cancer (N=normal, T=tumor; data obtained from TCGA data portal). LIHC=Hepatocellular Carcinoma (N=50, T=394).

a-c: Data is represented as log2 RSEM. Significance was calculated by unpaired, two-tailed t-test with *, $P<0.05$; **, $P<0.01$; ***, $P<0.001$.

Supplementary Figure 7

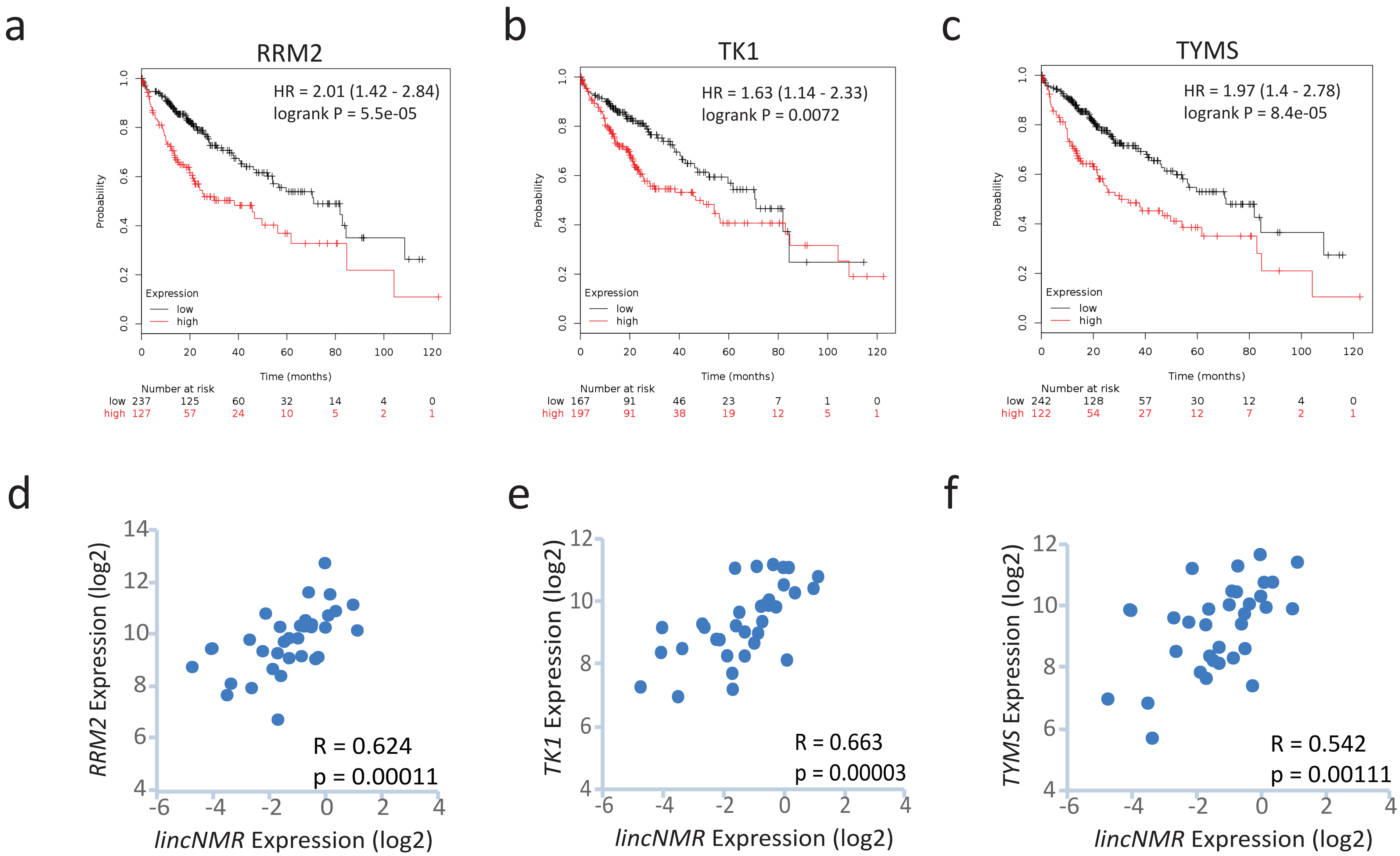

Supplementary Figure 7

Impact of *lincNMR* target proteins on survival and correlation with *lincNMR* in liver cancer

a. Kaplan-Meier plots of liver cancer patients stratified by RRM2 expression levels

b. Kaplan-Meier plots of liver cancer patients stratified by TK1 expression levels

c. Kaplan-Meier plots of liver cancer patients stratified by TYMS expression levels

d. Correlation plots: *lincNMR* expression correlates with *RRM2* mRNA expression in liver cancer patients (indicated: log2 expression)

e. Correlation plots: *lincNMR* expression with *TK1* mRNA expression in liver cancer patients (indicated: log2 expression)

f. Correlation plots: *lincNMR* expression with *TYMS* mRNA expression in liver cancer patients (indicated: log2 expression)

a-c: Kaplan-Meier plots were obtained from the KM plotter where survival plots were generated from the TCGA liver cancer patient dataset (LIHC) and significance was calculated using the log-rank test.

d-f: Correlation plots between *lincNMR* and *RRM2*, *TK1* and *TYMS* expression were obtained from TANRIC portal based on data derived from the TCGA liver cancer (LIHC) cohort.
