# Supplementary material for: The lncRNA lincNMR regulates nucleotide metabolism via a YBX1 - RRM2 axis in cancer
Source: Nat Commun. 2020 Jun 25;11:3214. doi: 10.1038/s41467-020-17007-9 (PMC7316977; doi:10.1038/s41467-020-17007-9)
Supplement: Supplementary file 2 — Description of Additional Supplementary Files [file 41467_2020_17007_MOESM2_ESM.pdf]

Description of Additional Supplementary Files

File Name: Supplementary Data 1
Description: List of lncRNAs screened for expression in nine liver cancer cell lines

File Name: Supplementary Data 2
Description: List of candidates identified in lincNMR RAP-MS

File Name: Supplementary Data 3
Description: Sequences of all siPOOLs used in this study

File Name: Supplementary Data 4
Description: Sequences of all RT-qPCR primers used in this study

File Name: Supplementary Data 5
Description: List of antibodies and dilutions used

File Name: Supplementary Data 6
Description: List of RACE primers used in this study

File Name: Supplementary Data 7
Description: List of RAP-MS probes used for lincNMR pulldown

File Name: Supplementary Data 8
Description: List of primers used for YBX1 ChIP

File Name: Supplementary Data 9
Description: Sequences of primers used for luciferase assay constructs

File Name: Supplementary Data 10
Description: Sequences of lincNMR cloning and mutagenesis primers used for generating YBX1 binding site mutations
