# Supplementary material for: The lncRNA lincNMR regulates nucleotide metabolism via a YBX1 - RRM2 axis in cancer
Source: Nat Commun. 2020 Jun 25;11:3214. doi: 10.1038/s41467-020-17007-9 (PMC7316977; doi:10.1038/s41467-020-17007-9)
Supplement: Supplementary file 13 — Reporting Summary [file 41467_2020_17007_MOESM13_ESM.pdf]

nature research

Corresponding author(s): Sven Diederichs

Last updated by author(s): May 29, 2020

Reporting Summary

Nature Research wishes to improve the reproducibility of the work that we publish. This form provides structure for consistency and transparency in reporting. For further information on Nature Research policies, see Authors & Referees and the Editorial Policy Checklist.

Statistics

For all statistical analyses, confirm that the following items are present in the figure legend, table legend, main text, or Methods section.

n/a	Confirmed	
☐	☒	The exact sample size (*n*) for each experimental group/condition, given as a discrete number and unit of measurement
☐	☒	A statement on whether measurements were taken from distinct samples or whether the same sample was measured repeatedly
☐	☒	The statistical test(s) used AND whether they are one- or two-sided<br>*Only common tests should be described solely by name; describe more complex techniques in the Methods section.*
☒	☐	A description of all covariates tested
☐	☒	A description of any assumptions or corrections, such as tests of normality and adjustment for multiple comparisons
☐	☒	A full description of the statistical parameters including central tendency (e.g. means) or other basic estimates (e.g. regression coefficient) AND variation (e.g. standard deviation) or associated estimates of uncertainty (e.g. confidence intervals)
☐	☒	For null hypothesis testing, the test statistic (e.g. *F*, *t*, *r*) with confidence intervals, effect sizes, degrees of freedom and *P* value noted<br>*Give P values as exact values whenever suitable.*
☒	☐	For Bayesian analysis, information on the choice of priors and Markov chain Monte Carlo settings
☒	☐	For hierarchical and complex designs, identification of the appropriate level for tests and full reporting of outcomes
☐	☒	Estimates of effect sizes (e.g. Cohen's *d*, Pearson's *r*), indicating how they were calculated

Our web collection on statistics for biologists contains articles on many of the points above.

Software and code

Policy information about availability of computer code

Data collection	n/a
Data analysis	StepOne Software v2.3<br>Perseus, Max Planck Institute of Biochemistry, Munich Germany<br>GraphPad Prism 8 GraphPad Software, San Diego, USA<br>Flow Jo v10, FlowJo, LLC<br>Image J- Image analysis software<br><br>Online Tools:<br>RBPMap: http://rbpmap.technion.ac.il/<br>TANRIC Data Portal: https://www.tanric.org/<br>KM Plotter: https://kmplot.com/<br>Panther Over-representation test: http://www.pantherdb.org/

For manuscripts utilizing custom algorithms or software that are central to the research but not yet described in published literature, software must be made available to editors/reviewers. We strongly encourage code deposition in a community repository (e.g. GitHub). See the Nature Research guidelines for submitting code & software for further information.

Data

Policy information about availability of data

All manuscripts must include a data availability statement. This statement should provide the following information, where applicable:

- Accession codes, unique identifiers, or web links for publicly available datasets
- A list of figures that have associated raw data
- A description of any restrictions on data availability

Sequencing data from 5' and 3' RACE experiment supporting both lincNMR isoforms have been deposited at Genbank with accession numbers MK652436 and MK652437. Source data for figures shown in this study are available upon request if not available in supplementary data and in the attached source data files. The source data underlying figures 4c,e and supplementary figures 4e,f, 2i as well as figures 1a-d,f,g, 2a,b,d, 3c-g, 4d,f, 5a,b,d,f, 6d,e, 7a-d,f and supplementary figures 1e,f, 2a-h, 3a-d, 4c,g,h, 5b-e, 6a-c are provided as Source Data files.

Field-specific reporting

Please select the one below that is the best fit for your research. If you are not sure, read the appropriate sections before making your selection.

☒ Life sciences ☐ Behavioural & social sciences ☐ Ecological, evolutionary & environmental sciences

For a reference copy of the document with all sections, see nature.com/documents/nr-reporting-summary-flat.pdf

Life sciences study design

All studies must disclose on these points even when the disclosure is negative.

Sample size	All experiments were executed in independent biological replicates and individual replicate numbers are given in the figure legends. Since this is not a clinical study or involves human or animal samples other than from public resources, a further sample size calculation is not applicable.
Data exclusions	For the publically available human cancer datasets, all available samples were used and none excluded.
Replication	All biological experiments were repeated in independent biological replicates. Precise numbers of biological replicates are given in the figure legends.
Randomization	Since this is not a clinical study, no randomization was necessary.
Blinding	Since this is not a clinical study, no blinding was necessary.

Reporting for specific materials, systems and methods

We require information from authors about some types of materials, experimental systems and methods used in many studies. Here, indicate whether each material, system or method listed is relevant to your study. If you are not sure if a list item applies to your research, read the appropriate section before selecting a response.

Materials & experimental systems

n/a	Involved in the study	
☐	☒	Antibodies
☐	☒	Eukaryotic cell lines
☒	☐	Palaeontology
☒	☐	Animals and other organisms
☒	☐	Human research participants
☒	☐	Clinical data

Methods

n/a	Involved in the study	
☒	☐	ChIP-seq
☐	☒	Flow cytometry
☒	☐	MRI-based neuroimaging

Antibodies

Antibodies used

Antibody Name Dilution WB Company Cat. No MW (kDa) Host
GAPDH 1:20000 Millipore #MAB374 37 mouse
Vinculin 1:1000 Santa Cruz sc-25336 116 mouse
RRM2 5 µg/mL Abcam ab57653 30(45) mouse
TYMS 1:2000 Abcam ab168853 36 rabbit
TK1 1:5000 Abcam ab76495 25 rabbit
YBX1 1:1000 Abcam ab12148 36 (50) rabbit
anti-HA 1:1000 Covance MMS-101P - mouse
anti-Flag-M2 1:1000 Sigma Aldrich 1804 - mouse

	anti-Flag magnetic beads - Sigma Aldrich M8823 - -<br>HuR (3A2); 1:500; Santa Cruz; sc-5621; 36 kDa; mouse<br>p21; 1:1000; Cell Signaling Technologies; 2947; 21 kDa; rabbit<br>p16; 1:1000; Abcam; ab51243; 16 kDa; rabbit<br>Phospho-p53 (Ser15); 1:1000; Cell Signaling Technologies; 9284P; 53 kDa; rabbit<br>P53; 1:2000; BD Pharmingen; 554293; 53 kDa; mouse<br>pRb1; 1:250; BD Pharmingen; 554136; 110; mouse<br>YBX1; (Used for In vitro RNA Affinity Purification) 1:1000; Abcam ; ab76149 ; 36 (50) kDa; rabbit<br>anti-mouse HRP-conjugated 2°Ab; 1:2500; Jackson ImmunoResearch Laboratories; 115-035-003<br>anti-rabbit HRP-conjugated 2°Ab; 1:2500; Jackson ImmunoResearch Laboratories; 111-035-144
Validation	All antibodies were used ad per manufacturer's recommendations. Dilutions used for the cell lines used is provided in Supplementary Data 5

Eukaryotic cell lines

Policy information about cell lines

Cell line source(s)	Liver Cancer Cell lines (HLE, HLF, SNU-387, FLC-4) used in this study were obtained from PD. Dr. Kai Breuhahn (Institute of Pathology) and are commercially available from ATCC; breast (MCF-7, KPL-1, T47D) and lung (A549, NCI-H460, NCI-H1299) cancer cell lines were purchased from ATCC.
Authentication	All cell lines used in this study were authenticated using cell line authentication service from Multiplexion, Heidelberg Germany using SNP based authentication approach.
Mycoplasma contamination	All cell lines were tested for mycoplasma contamination every three months using PCR Mycoplasma Test Kit I/C from PromoCell (Catalogue No. PK-CA91-1096) and were found mycoplasma negative.
Commonly misidentified lines<br>(See ICLAC register)	No commonly misidentified cell lines were used in this study.

Flow Cytometry

Plots

Confirm that:

☒ The axis labels state the marker and fluorochrome used (e.g. CD4-FITC).

☒ The axis scales are clearly visible. Include numbers along axes only for bottom left plot of group (a 'group' is an analysis of identical markers).

☒ All plots are contour plots with outliers or pseudocolor plots.

☒ A numerical value for number of cells or percentage (with statistics) is provided.

Methodology

Sample preparation	72 h post treatment with siPOOLs, cells were trypsinized and fixed in 70% Ethanol overnight at -20°C. Fixed cells were pelleted and washed with 1X PBS. After washing, cells were resuspended in 1X PBS containing 100 µg/ml RNase A (Sigma, 10109169001) and incubated at 37°C for 30 minutes. Post RNase treatment, cells were stained with 100 µg/ml Propidium Iodide (Sigma-Aldrich, P4170).
Instrument	BD FACSCanto II Flow Cytometer
Software	FlowJo v10 software.
Cell population abundance	Not applicable since only single stain (PI) used.
Gating strategy	After FSC/SSC gating as shown in source data file (.pptx), population analyzed using Cell Cycle Platform (Flow Jo) with PI channel on x-axis to get percentage of cells in G0/G1, S and G2/M phases.

☒ Tick this box to confirm that a figure exemplifying the gating strategy is provided in the Supplementary Information.
